# Ring-Opening Reaction of 1-Phospha-2-Azanorbornenes via P-N Bond Cleavage and Reversibility Studies

**DOI:** 10.3390/molecules28207163

**Published:** 2023-10-19

**Authors:** Kyzgaldak Ramazanova, Anna Karina Müller, Peter Lönnecke, Oldamur Hollóczki, Barbara Kirchner, Evamarie Hey-Hawkins

**Affiliations:** 1 Institute of Inorganic Chemistry, Faculty of Chemistry and Mineralogy, Leipzig University, Johannisallee 29, 04103 Leipzig, Germany; kyzgaldak.ramazanova@yahoo.com (K.R.); loenneck@rz.uni-leipzig.de (P.L.); 2 Mulliken Center for Theoretical Chemistry, Institute for Physical and Theoretical Chemistry, Beringstr. 4, 53115 Bonn, Germany; mueller@thch.uni-bonn.de (A.K.M.); kirchner@thch.uni-bonn.de (B.K.); 3 Department of Physical Chemistry, Faculty of Science and Technology, University of Debrecen, Egyetem ter 1, H-4010 Debrecen, Hungary; holloczki@gmail.com

**Keywords:** chirality, phospholes, racemates, reversibility, ring-opening reaction

## Abstract

The reactive P-N bond in 1-phospha-2-azanorbornenes is readily cleaved by simple alcohols to afford *P*-chiral 2,3-dihydrophosphole derivatives as a racemic mixture. The isolation of the products was not possible due to the reversibility of the reaction, which could, however, be stopped by sulfurization of the phosphorus atom, thus efficiently blocking the lone pair of electrons, as exemplified for **6b** yielding structurally characterized **8b**. Additionally, the influence of the substituent in the α position to the phosphorus atom (H, Ph, 2-py, CN) on the reversibility of the reaction was studied. Extensive theoretical calculations for understanding the ring-closing mechanism suggested that a multi-step reaction with one or more intermediates was most probable.

## 1. Introduction

**Scheme 1:**
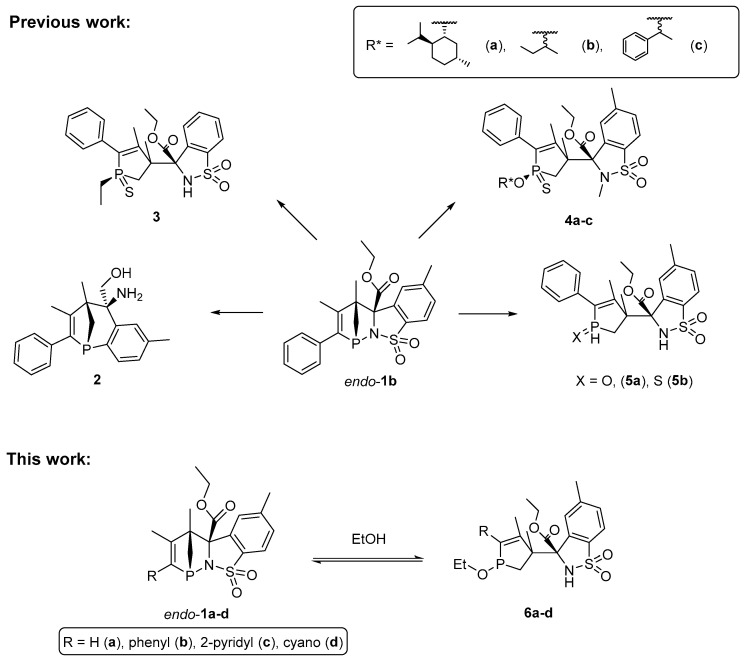
P-N bond cleavage reactions of PANs.

Phospholes are versatile starting materials for the production of phosphorus heterocyclic compounds [1,2,3,4,5,6]. The tautomerization of poorly aromatic 1*H*-phospholes leads to 2*H*-phospholes [7], which readily undergo hetero-Diels–Alder cycloaddition reactions with various dienophiles [8,9,10,11,12,13,14]. Thus, the stereoselective phospha-aza Diels–Alder reaction between 2*H*-phospholes and *N*-sulfonyl-α-iminoester affords 1-phospha-2-azanorbornenes (PANs) [9]. The cycloaddition reaction was successfully extended to a variety of previously reported P-substituted 2*H*-phospholes [15,16,17] to give a range of diastereomeric α-substituted PANs (*endo*-**1a**–**d**, Scheme 1) via a sigmatropic shift of the substituent at higher temperatures [7,18]. The general formation and properties of compounds containing phosphorus–nitrogen bonds have been extensively studied [19,20,21,22,23,24,25,26,27], such as cleavage reactions [28,29,30,31,32]. Thus, the P-N bond of PAN *endo*-**1b** can be cleaved by achiral nucleophiles, namely H_2_O, H_2_S, and EtMgBr, to give 2,3-dihydrophosphole derivatives (**5a,b** or **3**, Scheme 1). Enantiopure lithium alkoxides have also been used as powerful nucleophiles to afford diastereomeric mixtures of 1-alkoxy-2,3-dihydrophospholes (**4a**–**c**, Scheme 1), as well as enantiopure compounds obtained via the crystallization of the diastereomers (**4b**,**c**, Scheme 1) [33]. Furthermore, the reduction of PAN *endo*-**1b** with lithium aluminum hydride yielded a seven-membered phosphorus heterocycle (**2**, Scheme 1) [34].

All the abovementioned reactions were conducted with *endo*-**1b** only, while the reactivity of further PANs has remained largely unexplored. Herein, we report the P-N bond cleavage of PANs *endo*-**1a**–**d** (Scheme 1) with ethanol, resulting in the reversible formation of 1-ethoxy-2,3-dihydrophospholes **6a**–**d**, thus hampering the isolation of the final products. This behavior was already observed during the ring-opening reaction of *endo*-**1b** with H_2_O and alkoxides [9,33]. Consequently, the nature of this reversibility and its possible mechanism were studied.

## 2. Results and Discussion

### 2.1. P-N Bond Cleavage of endo-***1b*** with Alcohols

**Scheme 2:**
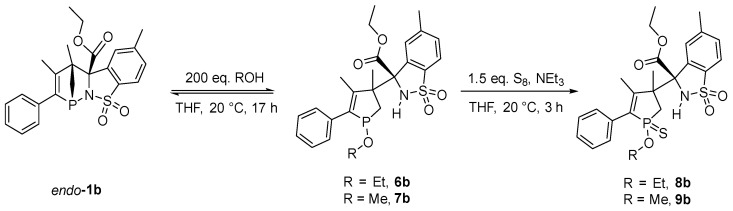
Synthesis of **8b** and **9b**. All chiral compounds presented here are racemic mixtures. For clarity, always only one enantiomer of each compound is shown.

**Scheme 3:**
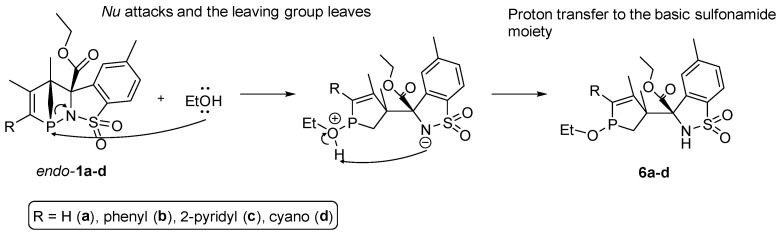
Proposed S_N_2 mechanism of the ring-opening reaction of PANs with EtOH.

Test reactions with ethanol as a nucleophile in the P-N bond cleavage were conducted with *endo*-**1b** (Scheme 2). Depending on the amounts of EtOH used, no product (5 eq. EtOH, Supplementary Materials, Figure S11), small amounts of **6b** (10 or 20 eq. EtOH), or a 1:1 ratio of *endo*-**1b**:**6b** (200 eq. EtOH, Scheme 2) were observed (determined using ^31^P{^1^H} NMR spectroscopy in C_6_D_6_). We assume the reaction occurs in an S_N_2 fashion, as shown in Scheme 3. A singlet at 141.7 ppm (cf. *endo*-**1b** 57 ppm), which is in the typical range for P^III^-O compounds, is assigned to the product **6b**, implying P-N bond cleavage and P-O bond formation [35]. When 20 or 200 eq. EtOH was employed, a second singlet appeared at 135.7 ppm, suggesting the formation of another diastereomer, as the small nucleophile can attack the P-N bond in *endo*-**1b** from different sides (ratio 4.5:1, according to ^31^P{^1^H} NMR spectroscopy).

All attempts to isolate the product **6b** failed. When the solvent was evaporated in vacuum, the ring-closing reaction took place, yielding predominantly *endo*-**1b**. A similar behavior was already observed for the ring-opening reaction of *endo*-**1b** with H_2_O and alkoxides [9,33].

Similarly, the P-N bond cleavage reaction in *endo*-**1b** with 200 eq. methanol also afforded two isomers of product **7b** in a 3:1 ratio, as confirmed by ^31^P{^1^H} NMR spectroscopy (C_6_D_6_) (145.3 and 140 ppm). Interestingly the second isomer was only produced when small nucleophiles were used, corroborating the logical dependence of the nucleophile size and number of products. Thus, in P-N bond cleavage reactions with bulky lithium alkoxides, only one isomer was formed [33]. The P-N cleavage reaction of *endo*-**1b** with MeOH is also reversible and undergoes a ring-closing reaction to form *endo*-**1b** when the reaction mixture is concentrated in vacuo.

As the lone pair of electrons at phosphorus in **6b** and **7b** is involved in the ring-closing reaction, the sulfur protection of **6b** and **7b** stopped the reversibility (Scheme 2) and allowed for a facile isolation of the **8b** and **9b** products via crystallization. Single crystals of **8b** and **9b** suitable for X-ray crystallography were obtained by dissolving **8b** or **9b** in hot isopropanol, followed by cooling at −25 °C overnight.

**Figure 1:**
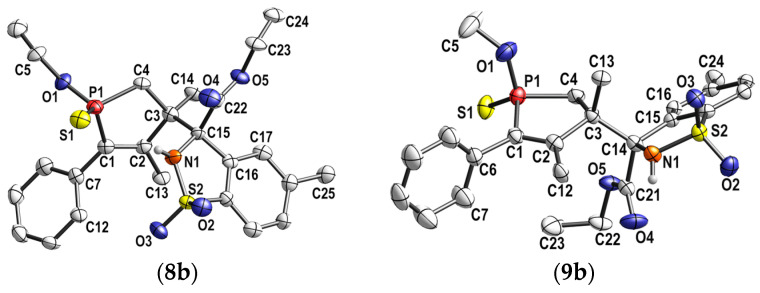
Molecular structures of **8b** and **9b**. Ellipsoids at 50% probability. Except NH, hydrogen atoms are omitted for clarity. For **8b**, the second symmetry-independent molecule of the asymmetric unit is shown in the Supplementary Materials, Figure S18.

In the structurally characterized compounds **8b** and **9b** (Figure 1), the phosphorus atom has a distorted tetrahedral environment. The P-O bond lengths are in the range from 158.8 to 159.5 pm, which corresponds to a P-O single bond. Consistent with the literature, the P=S double bonds in both compounds (191.5–193.8 pm) are in the expected ranges [36].

### 2.2. Reversibility Studies

In order to study the nature of the reversibility of the P-N bond cleavage reactions with ethanol, we included previously reported 1-phospha-2-azanorbornenes (PANs) *endo*-**1a**,**c**,**d** (Scheme 1), which have different substituents in the α position to the phosphorus atom. These PANs are also racemic mixtures resulting in racemic products. The enantiomers were never isolated. Firstly, *endo*-**1c** was reacted with 200 eq. of EtOH to afford **6c** in a ratio of 1:3 (**6c**:*endo*-**1c**, Supplementary Materials, Figure S12). Concentrating the THF solution in vacuum at 20 °C resulted in ring closing mainly yielding the starting material. To verify if the size of the substituent in the α position to phosphorus in PANs correlates with the rate of conversion and reversibility of the reaction, we conducted a P-N bond cleavage reaction with *endo*-**1a**, the smallest PAN bearing a hydrogen atom in the α position, with 200 eq. of EtOH (Supplementary Materials, Figure S10) overnight at room temperature. The ^31^P{^1^H} NMR spectrum of the reaction mixture showed a 1:1 ratio of *endo*-**1a** and **6a**. Interestingly, the ring-closing reaction took place only to some extent when the solution was concentrated in vacuum at 20 °C, affording a ratio of 2:1 of *endo-***1a** to product **6a**. Finally, *endo-***1d** containing a nitrile group in the α position was reacted with 200 eq. EtOH. The reaction proceeded with the highest conversion (1:8 for *endo*-**1d**:**6d**) compared to all the PANs, as identified using ^31^P{^1^H} NMR spectroscopy. Surprisingly, the ring-closing reaction did not take place when concentrating the solution in vacuum, even at 60 °C, suggesting that there is no equilibrium between *endo*-**1d** and **6d** (Supplementary Materials, Figure S13).

Density functional theory calculations were performed to obtain the relative free energies ΔG of the ring-closing reactions (Table 1).

These values support the experimental findings and show that the equilibrium is shifted towards the reactants for the reaction of *endo*-**1b**,**c** with EtOH, while in the reaction of *endo*-**1a**,**d** with EtOH, the equilibrium is shifted towards the products. This trend seems to be largely independent of the size of the α substituents of the PANs, as the ^31^P{^1^H} NMR spectroscopic reversibility studies did not show any logical dependance.

The reversibility of these reactions could, however, be influenced by the electronic environment around the P atom governed by the substituents. Therefore, we considered the frontier orbitals of the products **6a**–**d** being located either on P or N for a nucleophilic attack of HOMO to LUMO, thus facilitating the ring-closing reaction (for frontier orbitals calculations of **6a**–**d** see Supplementary Materials, Figure S17). Most of the HOMO contributions in **6a**–**d** come from P atomic orbitals, as well as the substituents in the α position. The LUMO contributions in **6a**–**d** are mostly scattered throughout the molecule, mainly on the backbone of the sulfonamide moiety. The largest HOMO-LUMO energy gap was calculated for *endo*-**1a** and **6a** (Table 2). However, the calculation of the frontier orbitals did not give a clear explanation for the abovementioned reactivity trends.

**Figure 2:**
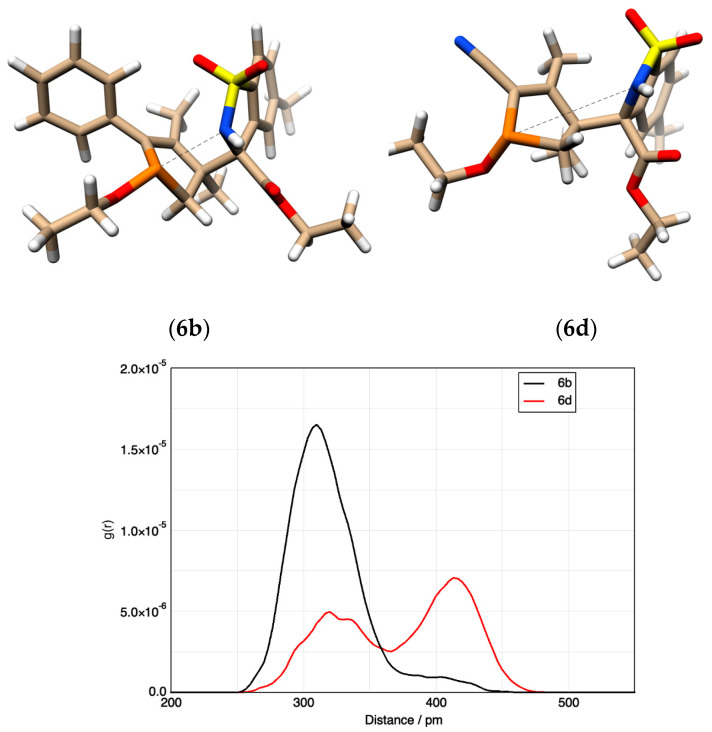
The structures of **6b** (**top left**) and **6d** (**top right**) after the AIMD simulations with the P···N distance visualized as a dashed line and the radial distribution functions of the P···N distance (**bottom**).

To understand the mechanism of the back-reaction, we searched for transition states and intermediates using density functional theory. However, we were not able to obtain a reasonable transition state between the reactants (*endo*-**1a**–**d**) and products (**6a**–**d**). This could indicate a multi-step reaction with one or more intermediates. Since EtOH was used in a large excess in the P-N bond cleavage reaction, an additional structure containing the product (**6a**–**d**) and a second ethanol molecule was calculated. However, this structure was found to be unstable, suggesting that the excess of EtOH does not play a role in the back-reaction. Furthermore, ab initio molecular dynamics (AIMD) simulations of the products **6b** and **6d** in ethanol were performed. Interesting developments in the simulations of the product structures **6b** (fully reversible) and **6d** (completely irreversible) were found. While product **6b** looks as expected from the X-ray structure determination and DFT optimizations, the P···N distance in product **6d** shows a significant elongation after some simulation time (Figure 2).

In order to analyze the different behaviors of **6b** and **6d** in ethanol, the radial distribution functions (RDFs) of the P···N distance were calculated (Figure 2, bottom). In the case of **6b**, only one peak at about 310 pm with a small shoulder at longer distances can be seen, indicating that the P···N distance stays rather constant at shorter distances. The RDF of **6d** shows two broad peaks, with the smaller one at short distances around 320 pm and the slightly bigger peak at around 415 pm. Thus, the P···N distance in **6d** is, on average, elongated compared to the one in **6b**. This could explain why a back-reaction would not occur in the case of product **6d**.

## 3. Conclusions

In summary, we described the formation of racemic *P*-chiral 1-ethoxy-2,3-dihydrophosphole derivatives via the nucleophilic cleavage of the P-N bond in 1-phospha-2-azanorbornenes *endo*-**1a**–**d** with ethanol. The isolation of the products was not possible due to the reversibility of the reaction, which could, however, be stopped by sulfurization of the phosphorus atom, thus efficiently blocking the lone pair of electrons, as exemplified for **6b**, yielding structurally characterized **8b**. Additionally, the influence of the substituent in the α position to the phosphorus atom (*endo*-**1a**,**c**,**d**) was studied. Extensive theoretical calculations, conducted to understand the ring-closing mechanism, suggested that a multi-step reaction with one or more intermediates was most probable. Overall, the ring-closing reaction seemed to depend strongly on the electronic environment around the phosphorus atom, as a nitrile group in the α position hindered the reversibility. Although the exact mechanism of the ring-closing reaction is not understood, it could be shown that small and electron-withdrawing groups located at the α position to the phosphorus atom gave access to thermodynamically stable 1-ethoxy-dihydrophosphole derivatives. Further mechanistic studies are underway.

## 4. Materials and Methods

### 4.1. General Information

All air-sensitive reactions were carried out under dry high-purity nitrogen using the standard Schlenk technique. THF was degassed and distilled from potassium. EtOH and MeOH were degassed and kept over activated molecular sieves. The NMR spectra were recorded with a Bruker Avance DRX 400 spectrometer (^1^H NMR 400.13 MHz, ^13^C NMR 100.63 MHz, and ^31^P NMR 161.98 MHz, Bruker, Rheinstetten, Germany). ^13^C{^1^H} NMR spectra were recorded as APT spectra. The assignment of the chemical shifts and configurations was performed using the COSY and HSQC techniques. TMS was used as the internal standard in the ^1^H NMR spectra and all the other nuclei spectra were referenced to TMS using the Ξ-scale [37]. The numbering scheme of **6d**, **8b**, and **9b** is given in the Supplementary Materials. High-resolution mass spectra (HRMS; ESI) were measured using a Bruker Daltonics APEX II FT-ICR spectrometer (Billerica, MA, USA). IR spectra were obtained with an FTIR spectrometer (Nicolet iS5 FTIR by Thermo Scientific, Waltham, MA, USA) in the range of 400–4000 cm^−1^ in KBr. The compounds *endo*-**1a**–**d** were prepared according to the literature [9].

### 4.2. Synthesis

General procedure for the preparation of **8b** and **9b.**

Alcohol (EtOH: 3.16 mL; MeOH: 2.18 mL; 200 eq., 54 mmol) was added at room temperature to a solution of 120 mg (0.27 mmol) of *endo-***1b** in 5 mL of THF. The reaction mixture was stirred overnight followed by the addition of 13 mg (0.4 mmol) of elemental sulfur and 2 drops of NEt_3_. After stirring for 6 h, the solvent was removed in vacuo and the crude product was isolated by dissolving in hot *i*PrOH, followed by cooling at −25 °C overnight to give **8b** or **9b** as a white powder.

**8b**: 103 mg (73%)

^1^H NMR (400 MHz, CDCl_3_) δ 7.74–7.66 (m, 2H, H-aryl), 7.49–7.23 (m, 6H, H-aryl), 6.14 (bs, 1H, N-H), 4.43–4.30 (m, 2H, H-5b), 4.08–3.92 (m, 2H, H-12), 3.23–3.1 (m, 1H, H-4), 2.54 (s, 3H, H-8a), 2.18 (m, 1H, H-4), 1.69 (s, 3H, H-3a/2a), 1.56 (s, 3H, H-2a/3a), 1.41 (t, *J* = 7.1 Hz, 3H, H-5c), and 1.17 (t, 3H, H-13) ppm; ^13^C{^1^H} NMR (101 MHz, CDCl_3_) δ 169.2 (s, C-5a), 156.7 (d, *J* = 30.1 Hz, C-1 or C-2), 144.1 (s, C-8), 139.0 (d, *J* = 98.8 Hz, C-1/2), 134.9 (s, C-quart. aryl), 134.0 (s, C-quart. aryl), 132.7 (d, *J* = 9.8 Hz, C-quart. aryl), 129.2 (d, *J* = 4.2 Hz, C-1b or C-1c), 128.5 (s, C-1b or C-1c), 128.0 (s, C-1d), 127.7 (s, C-7), 121.7 (s, C-10), 73.5 (d, *J* = 10.1 Hz, C-5), 64.5 (s, C-5b), 61.7 (d, *J* = 6.6 Hz, C-12), 42.9 (d, *J* = 70.9 Hz, C-4), 24.7 (s, C-2a/3a), 22.2 (s, C-8a), 16.5 (d, *J* = 6.5 Hz, C-13), 15.7 (d, *J* = 17.2 Hz, C-2a/3a), and 14.2 (s, C-5c) ppm; ^31^P{^1^H} NMR (162 MHz, CDCl_3_) δ 104.7 (s) ppm; ^31^P NMR (162 MHz, CDCl_3_) δ 104.7 (m) ppm; HRMS (ESI+, MeCN), *m/z*: found: 520.1385; calc. for [M+H]^+^: 520.1376, found: 542.1223; calc. for [M+Na]^+^: 542.1195; found: 558.0936, calc. for [M+K]^+^: 558.0935; found: 1061.2525, calc. for [2M+Na]^+^: 1061.2498; and found: 1077.2222, calc. for [2M+K]^+^: 1077.2238; IR (KBr): *ṽ* = 3278 (m), 2978 (m), 1728 (s, C=O in ester), 1621 (w), 1596 (m), 1492 (w), 1473 (w), 1441 (w), 1409 (w), 1387 (m), 1292 (s), 1252 (s), 1230 (s), 1183 (s), 1148 (s), 1130 (s), 1105 (s), 1015 (s, O-C from P-O-C_2_H_5_ fragment), 964 (m), 949 (m), 922 (s, C-C from P-O-C_2_H_5_ fragment), 874 (s), 861 (s), 846 (m), 823 (s), 807 (s), 788 (m), 755 (s), 701 (s), 685 (s), 668 (s), 652 (s), 626 (s), 607 (s), 575 (s, possibly P=S), 548 (m), 514 (m), 495 (s), and 433 (s) cm^−1^.

**9b**: 94 mg (68%)

^1^H NMR (400 MHz, CDCl_3_) δ 7.67–7.59 (m, 2H, H-aryl), 7.39 (d, *J* = 8.0 Hz, 1H, H-aryl), 7.32 (m, 2H, H-aryl), 7.26–7.14 (m, 3H, H-aryl), 6.03 (bs, 1H, N-H), 4.30 (m, 2H, H-5b), 3.53 (d, *J* = 14.1 Hz, 3H, H-12), 3.10 (m, 1H, H-4), 2.47 (s, 3H, H-8a), 2.18–2.06 (m, 1H, H-4), 1.62 (s, 3H, H-2a/3a), 1.48 (s, 3H, H-3a/2a), and 1.34 (t, *J* = 7.1 Hz, 3H, H-5c) ppm; ^13^C{^1^H} NMR (101 MHz, CDCl_3_) δ 169.1 (s, C-5a), 156.9 (d, *J* = 29.8 Hz, C-1/2), 144.1 (C-8), 138.7 (d, *J* = 98.8 Hz, C-1/2), 134.4 (d, *J* = 90.3 Hz, C-quart.), 132.6 (d, *J* = 9.4 Hz, C-quart), 131.9 (s), 129.1 (d, *J* = 4.3 Hz), 128.5, 127.9 (d, *J* = 1.9 Hz), 127.6, 73.4 (d, *J* = 10.3 Hz), 64.5 (s, C-5b), 52.1 (d, *J* = 6.7 Hz, C-12), 41.9 (d, *J* = 71.0 Hz, C-4), 24.6 (s, C-2a/3a), 22.1 (s, C-8), 15.6 (d, *J* = 17.1 Hz, C-2a/3a), and 14.1 (s, C-5c) ppm; ^31^P{^1^H} NMR (162 MHz, CDCl_3_) δ 107.3 (s) ppm; ^31^P NMR (162 MHz, CDCl_3_) δ 107.3 (m) ppm; HRMS (ESI, MeCN/DCM), *m/z*: found: 520.1385, calc. for [M+H]^+^: 520.1376; found: 528.1059 calc. for [M+Na]^+^: 528.1039; found: 544.0793, calc. for [M+K]^+^: 544.0778; and found: 1033.2223, calc. for [2M+Na]^+^: 1033.2185; IR (KBr): *ṽ* = 3269 (m), 2978 (w), 2940 (w), 1715 (s, C=O in ester), 1616 (w), 1593 (m), 1487 (w), 1440 (m), 1409 (w), 1381 (m), 1365 (m), 1301 (s), 1249 (s, C-O in ester), 1184 (s), 1140 (s), 1114 (m), 1070 (s), 1033 (s, O-C from P-O-CH_3_ fragment), 1011 (s), 965 (s), 908 (m), 877 (w), 859 (s), 846 (m), 846 (m), 822 (w), 792 (w), 765 (s), 747 (s), 726 (s), 698 (s), 646 (s), 615 (m), 600 (m), 582 (m), 567 (s, P=S possibly), 538 (s), 521 (m), 498 (m), 473 (m), and 435 (m) cm^−1^.

### 4.3. Reversibility Studies

The reversibility experiments were conducted by adding 200 eq. of EtOH (20 eq. was used in the case of *endo*-**1d**) to a solution of the corresponding PAN in 3 mL of THF (50 mg, 0.137 mmol of *endo*-**1a** and 27 mmol, 1.6 mL of EtOH; 50 mg, 0.113 mmol of *endo*-**1b** and 23 mmol, 1.3 mL of EtOH; 50 mg, 0.113 mmol of *endo*-**1c** and 23 mmol, 1.3 mL of EtOH; 50 mg, 0.128 mmol of *endo*-**1d** and 2.6 mmol, 0.15 mL of EtOH) at room temperature. After stirring overnight, NMR samples were taken. The solutions were concentrated in vacuo followed by drying for one hour. Then, another NMR sample was taken to study the reversibility (Supplementary Materials, Figures S10–13).

### 4.4. Computational Details

The quantum chemical calculations were performed with the Orca program version 4.2.1. [38]. All structures were optimized using the B3LYP [39,40] functional with the D3 [41,42] dispersion correction with Becke-Johnson damping and the def2-TZVP [43] basis set. For the SCF cycle, a tight convergence criterium was chosen. To ensure that all optimized structures are minima on the potential energy surface, a subsequent frequency calculation was performed, where all the stationary points resulted in non-negative eigenvalues of the Hessians. The molecular orbitals were calculated at Hartree–Fock level of theory on the DFT-optimized geometries, employing the same level of theory as before.

Additional ab initio molecular dynamics simulations were performed. The initial configurations of the simulation boxes were set up with PACKMOL [44], each containing 128 molecules of ethanol and one molecule of **6b** and **6d**, respectively, using the experimental density of ethanol at 300 K [45]. The simulations were performed with CP2K version 5.1 [46] using periodic boundary conditions and applying the QUICKSTEP [47] module. The BLYP [40,48] functional and the corresponding BLYP Goedecker–Teter–Hutter pseudopotentials [49,50,51] for core electrons were applied for the DFT part in combination with the molecularly optimized double-zeta basis set (MOLOPT-DZVP-SR-GTH) [52]. To account for dispersion effects, the DFT-D3 correction was used. The simulation boxes were equilibrated for 5 ps at 350 K by applying a thermostat, and a subsequent production run was performed for 27.5 ps at 350 K. A density cutoff of 400 Ry was chosen, as well as a relative cutoff of 40. For the SCF, an accuracy threshold of 10^−6^ was applied. The simulations were performed in the canonical (NVT) ensemble using the Nosé-Hoover chain thermostats [53,54,55] and a time step of 0.5 fs. The resulting AIMD trajectories were analyzed using the TRAVIS program [56].

### 4.5. X-ray Crystallography Data

The data were collected on a Gemini diffractometer (Rigaku Oxford Diffraction) using Mo-Kα radiation and ω-scan rotation. Data reduction was performed with CrysAlisPro [57], including the program SCALE3 ABSPACK for empirical absorption correction. All structures were solved using dual-space methods with SHELXT [58] and the refinement was performed with SHELXL [59]. With the exception of hydrogen atoms at nitrogen, all H atoms were calculated on idealized positions using the riding model. The S=P-O-Me fragment of **9b** was disordered with a ratio of 0.866 (6):0.134 (6). Structure figures were generated with DIAMOND-4 [60].

CCDC deposition numbers 2291772 for **8b** and 2291773 for **9b** contain the supplementary crystallographic data for this paper. These data can be obtained free of charge via https://www.ccdc.cam.ac.uk/structures (or from the Cambridge Crystallographic Data Centre, 12 Union Road, Cambridge CB2 1EZ, UK; fax: (+44)1223-336-033; or deposit@ccdc.cam.uk).

## Figures and Tables

**Table 1:** ΔG values of the P-N bond cleavage reactions of *endo*-**1a**–**d** with EtOH.

Reaction	ΔG/kcal mol^−1^
*endo*-**1a** → **6a**	−0.2
*endo*-**1b** → **6b**	0.8
*endo*-**1c** → **6c**	0.4
*endo*-**1d** → **6d**	−1.1

**Table 2:** Calculated HOMO-LUMO gaps of the different reactants *endo*-**1a**–**d** and products **6a**–**d**.

Compound	Gap/eV	Compound	Gap/eV
*endo*-**1a**	12.0	**6a**	11.3
*endo*-**1b**	10.8	**6b**	10.5
*endo*-**1c**	10.4	**6c**	11.1
*endo*-**1d**	11.8	**6d**	11.2

## Data Availability

Not applicable.

## Supplementary Materials

The following supporting information can be downloaded at: https://www.mdpi.com/article/10.3390/molecules28207163/s1. NMR spectra and numbering scheme of **8b** and **9b**. P-N bond cleavage of *endo*-**1b** with EtOH. Reversibility studies; synthesis, NMR spectra and numbering scheme of **6b**. Theoretical calculations, xyz-structures, HOMO-LUMO orbitals. X-ray crystallography data.

## References

[1] Wang H., Li C., Geng D., Chen H., Duan Z., Mathey F. (2010). A new versatile route for the conversion of phospholes into phosphinines. Chem. Eur. J..

[2] Luo H., Wang J., Tian R., Duan Z. (2023). 2*H*-Phosphindole-Enabled Dearomatization and 4+2 Cycloaddition of (Hetero)Arenes. Chem. Eur. J..

[3] Duffy M.P., Delaunay W., Bouit P.-A., Hissler M. (2016). π-Conjugated phospholes and their incorporation into devices: Components with a great deal of potential. Chem. Soc. Rev..

[4] König N., Godínez-Loyola Y., Yang F., Laube C., Laue M., Lönnecke P., Strassert C.A., Hey-Hawkins E. (2023). Facile modification of phosphole-based aggregation-induced emission luminogens with sulfonyl isocyanates. Chem. Sci..

[5] Mathey F. (1988). The organic chemistry of phospholes. Chem. Rev..

[6] Johannsen T., Golz C., Alcarazo M. (2020). α-Cationic Phospholes: Synthesis and Applications as Ancillary Ligands. Angew. Chem. Int. Ed. Engl..

[7] Mathey F. (2004). Transient 2H-phospholes as powerful synthetic intermediates in organophosphorus chemistry. Acc. Chem. Res..

[8] Möller T., Wonneberger P., Sárosi M.B., Coburger P., Hey-Hawkins E. (2016). P-chiral 1-phosphanorbornenes: From asymmetric phospha-Diels-Alder reactions towards ligand design and functionalisation. Dalton Trans..

[9] Wonneberger P., König N., Kraft F.B., Sárosi M.B., Hey-Hawkins E. (2019). Access to 1-Phospha-2-azanorbornenes by Phospha-aza-Diels-Alder Reactions. Angew. Chem. Int. Ed. Engl..

[10] Toullec P., Ricard L., Mathey F. (2003). Hetero-Diels-Alder reactions of 2H-phospholes with aldehydes. J. Org. Chem..

[11] Jia S., Ma M., Li E.-Q., Duan Z., Mathey F. (2021). Design of 1-Phosphanorbornene Derivatives as Chiral Organocatalysts for Enantioselective (4 + 2) Annulation Reactions of γ-Benzyl Allenoates. Org. Lett..

[12] Gan Z., Zhi M., Han R., Li E.-Q., Duan Z., Mathey F. (2019). P-Stereogenic Phosphines Directed Copper(I)-Catalyzed Enantioselective 1,3-Dipolar Cycloadditions. Org. Lett..

[13] Zhang K., Zhang Q., Wei D., Tian R., Duan Z. (2021). Hetero-Diels–Alder reactions of 2*H*-phospholes with allenes: Synthesis and functionalization of 6-methylene-1-phosphanorbornenes. Org. Chem. Front..

[14] Robin F., Lelièvre S., Mercier F., Ricard L., Mathey F. (2002). 1-Phosphanorbornadienes in Enantioselective Catalysis. Phosphorus Sulfur Silicon Relat. Elem..

[15] Holand S., Mathey F. (1988). Introducing new phosphorus substituents in terminal phosphinidene complexes. An illustration with [(ethoxycarbonyl)phosphinidene]-, (tert-butoxyphosphinidene)-, and (fluorenylphosphinidene)pentacarbonyltungsten complexes. Organometallics.

[16] Holand S., Jeanjean M., Mathey F. (1997). A Straightforward Access to α-Functional Phospholide Ions. Angew. Chem. Int. Ed. Engl..

[17] Breque A., Mathey F., Savignac P. (1981). An Improved One-Pot Synthesis of Phospholes. Synthesis.

[18] Mathey F., Mercier F., Robin F., Ricard L. (1998). From 2*H*-phospholes to BIPNOR, a new efficient biphosphine for asymmetric catalysis. J. Organomet. Chem..

[19] Krishnamurthy S.S. (1994). Phosphazenes and Phosphazanes—The Nature of the P-N Bond. Phosphorus Sulfur Silicon Relat. Elem..

[20] Emsley J., Williams J.K. (1973). The phosphorus–nitrogen bond. Synthesis, characterization, and infrared studies of heterocyclic phosphoryl (phosphetan) amides. J. Chem. Soc. Dalton Trans..

[21] Arias A., Forniés J., Fortuño C., Ibáñez S., Martín A., Mastrorilli P., Gallo V., Todisco S. (2013). Addition of nucleophiles to phosphanido derivatives of Pt(III): Formation of P-C, P-N, and P-O bonds. Inorg. Chem..

[22] Marinetti A., Carmichael D. (2002). Synthesis and properties of phosphetanes. Chem. Rev..

[23] Alvarez M.A., Cuervo P.M., García M.E., Ruiz M.A., Vega P. (2022). P-C, P-N, and M-N Bond Formation Processes in Reactions of Heterometallic Phosphinidene-Bridged MoMn and MoRe Complexes with Diazoalkanes and Organic Azides to Build Three- to Five-Membered Phosphametallacycles. Inorg. Chem..

[24] Varadwaj A., Varadwaj P.R., Marques H.M., Yamashita K. (2022). Definition of the Pnictogen Bond: A Perspective. Inorganics.

[25] Bai Z., Song F., Wang H., Cheng W., Zhu S., Huang Y., He G., Chen G. (2022). Nitrene-Mediated P–N Coupling Under Iron Catalysis. CCS Chem..

[26] Ding T.-T., Qin C.-C., Gao D.-D., Li X.-J., Yan H., Liu R.-M., Zhao C.-Q., Nie S.-Z. (2019). Stereoselective formation of P-N bonds via coupling of H-P species with amines and the addition of Grignard reagents to chiral N-phosphinoylimines. Phosphorus Sulfur Silicon Relat. Elem..

[27] Albuerne I.G., Alvarez M.A., García M.E., García-Vivó D., Ruiz M.A., Vega P. (2020). P-N and N-Mo Bond Formation Processes in the Reactions of a Pyramidal Phosphinidene-Bridged Dimolybdenum Complex with Diazoalkanes and Organic Azides. Inorg. Chem..

[28] Misiura K., Silverton J.V., Stec W.J. (1985). Stereochemistry of phosphorus-nitrogen bond cleavage. First crystal and structural assignment in cyclic phosphoramidofluoridates. J. Org. Chem..

[29] Gurnani C., Đorđević N., Muthaiah S., Dimić D., Ganguly R., Petković M., Vidović D. (2015). Extending the chemistry of carbones: P-N bond cleavage via an S(N)2′-like mechanism. Chem. Commun..

[30] Lesiak K., Stec W.J. (1978). The Stereochemistry of P-N Bond Cleavage in the Staudinger-Wittig-type Reaction of 2-Anilido-3,4-dimethyl-5-phenyl-1,3,2-oxazaphospholidine-2-thiones. Z. Naturforsch. B.

[31] Han Z.S., Goyal N., Herbage M.A., Sieber J.D., Qu B., Xu Y., Li Z., Reeves J.T., Desrosiers J.-N., Ma S. (2013). Efficient asymmetric synthesis of P-chiral phosphine oxides via properly designed and activated benzoxazaphosphinine-2-oxide agents. J. Am. Chem. Soc..

[32] Greenhalgh R., Blanchfield J.R. (1966). The cleavage of phosphorus to nitrogen bonds with hydrogen fluoride. Can. J. Chem..

[33] Ramazanova K., Lönnecke P., Hey-Hawkins E. (2023). Facile Synthesis of Enantiomerically Pure P-Chiral 1-Alkoxy-2,3-dihydrophospholes via Nucleophilic P-N Bond Cleavage of a 1-Phospha-2-azanorbornene. Chem. Eur. J..

[34] Wonneberger P., König N., Sárosi M.B., Hey-Hawkins E. (2021). Reductive Rearrangement of a 1-Phospha-2-azanorbornene. Chem. Eur. J..

[35] Kühl O. (2009). Phosphorus-31 NMR Spectroscopy: A Concise Introduction for the Synthetic Organic and Organometallic Chemist.

[36] Allen F.H., Kennard O., Watson D.G., Brammer L., Orpen A.G., Taylor R. (1987). Tables of bond lengths determined by X-ray and neutron diffraction. Part 1. Bond lengths in organic compounds. J. Chem. Soc. Perkin Trans. 2.

[37] Harris R.K., Becker E.D., Cabral de Menezes S.M., Goodfellow R., Granger P. (2002). NMR Nomenclature: Nuclear Spin Properties and Conventions for Chemical Shifts. IUPAC Recommendations 2001. Solid State Nucl. Magn. Reson..

[38] Neese F. (2012). The ORCA program system. WIREs Comput. Mol. Sci..

[39] Becke A.D. (1993). Density-functional thermochemistry. III. The role of exact exchange. J. Chem. Phys..

[40] Lee C., Yang W., Parr R.G. (1988). Development of the Colle-Salvetti correlation-energy formula into a functional of the electron density. Phys. Rev. B Condens. Matter.

[41] Grimme S., Antony J., Ehrlich S., Krieg H. (2010). A consistent and accurate ab initio parametrization of density functional dispersion correction (DFT-D) for the 94 elements H-Pu. J. Chem. Phys..

[42] Grimme S., Ehrlich S., Goerigk L. (2011). Effect of the damping function in dispersion corrected density functional theory. J. Comput. Chem..

[43] Weigend F., Ahlrichs R. (2005). Balanced basis sets of split valence, triple zeta valence and quadruple zeta valence quality for H to Rn: Design and assessment of accuracy. Phys. Chem. Chem. Phys..

[44] Martínez L., Andrade R., Birgin E.G., Martínez J.M. (2009). PACKMOL: A package for building initial configurations for molecular dynamics simulations. J. Comput. Chem..

[45] Khattab I.S., Bandarkar F., Fakhree M.A.A., Jouyban A. (2012). Density, viscosity, and surface tension of water+ethanol mixtures from 293 to 323K. Korean J. Chem. Eng..

[46] Kühne T.D., Iannuzzi M., Del Ben M., Rybkin V.V., Seewald P., Stein F., Laino T., Khaliullin R.Z., Schütt O., Schiffmann F. (2020). CP2K: An electronic structure and molecular dynamics software package–Quickstep: Efficient and accurate electronic structure calculations. J. Chem. Phys..

[47] VandeVondele J., Krack M., Mohamed F., Parrinello M., Chassaing T., Hutter J. (2005). Quickstep: Fast and accurate density functional calculations using a mixed Gaussian and plane waves approach. Comput. Phys. Commun..

[48] Becke A.D. (1988). Density-functional exchange-energy approximation with correct asymptotic behavior. Phys. Rev. A Gen. Phys..

[49] Goedecker S., Teter M., Hutter J. (1996). Separable dual-space Gaussian pseudopotentials. Phys. Rev. B Condens. Matter.

[50] Hartwigsen C., Goedecker S., Hutter J. (1998). Relativistic separable dual-space Gaussian pseudopotentials from H to Rn. Phys. Rev. B Condens. Matter.

[51] Krack M. (2005). Pseudopotentials for H to Kr optimized for gradient-corrected exchange-correlation functionals. Theor. Chem. Acc..

[52] VandeVondele J., Hutter J. (2007). Gaussian basis sets for accurate calculations on molecular systems in gas and condensed phases. J. Chem. Phys..

[53] Nosé S. (1984). A unified formulation of the constant temperature molecular dynamics methods. J. Chem. Phys..

[54] Nosé S. (1984). A molecular dynamics method for simulations in the canonical ensemble. Mol. Phys..

[55] Martyna G.J., Klein M.L., Tuckerman M. (1992). Nosé–Hoover chains: The canonical ensemble via continuous dynamics. J. Chem. Phys..

[56] Brehm M., Thomas M., Gehrke S., Kirchner B. (2020). TRAVIS-A free analyzer for trajectories from molecular simulation. J. Chem. Phys..

[57] Rigaku Corporation (1995–2023). CrysAlisPro Software System.

[58] Sheldrick G.M. (2015). SHELXT–integrated space-group and crystal-structure determination. Acta Crystallogr. A Found. Adv..

[59] Sheldrick G.M. (2015). Crystal structure refinement with SHELXL. Acta Crystallogr. C Struct. Chem..

[60] Crystal Impact GbR.

