# Supplementary material for: Ring-Opening Reaction of 1-Phospha-2-Azanorbornenes via P-N Bond Cleavage and Reversibility Studies
Source: Molecules. 2023 Oct 19;28(20):7163. doi: 10.3390/molecules28207163 (PMC10609391; doi:10.3390/molecules28207163)
Supplement: Supplementary file 1 [file molecules-28-07163-s001.zip › molecules-2647790-supplementary.pdf]

Ring-Opening Reaction of 1-Phospha-2-Azanorbornenes via P-N Bond Cleavage and Reversibility Studies

Kyzgaldak Ramazanova [1], Anna Karina Müller [2], Peter Lönnecke [1], Oldamur Hollóczki [3], Barbara Kirchner [2] and Evamarie Hey-Hawkins [1,*]

[1] Institute of Inorganic Chemistry, Faculty of Chemistry and Mineralogy, Leipzig University, Johannisallee 29, 04103 Leipzig, Germany

[2] Mulliken Center for Theoretical Chemistry, Institute for Physical and Theoretical Chemistry, Beringstr. 4, 53115 Bonn, Germany

[3] Department of Physical Chemistry, Faculty of Science and Technology, University of Debrecen, Egyetem ter 1, H-4010 Debrecen, Hungary

*Correspondence: hey@uni-leipzig.de

Contents

1. NMR spectra and numbering scheme of **8b** and **9b** 3
2. P–N bond cleavage of *endo*-**1b** with EtOH 7
3.1. Reversibility studies 8
3.2. Synthesis, NMR spectra and numbering scheme of **6b** 10
4. Theoretical calculations 12
4.1. xyz-structures 12
4.2. HOMO-LUMO orbitals 22
5. X-ray crystallography data 23

1. NMR spectra and numbering scheme of **8b** and **9b**

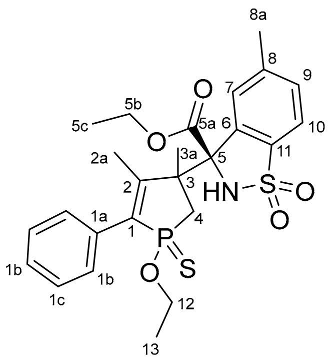

(**8b**)

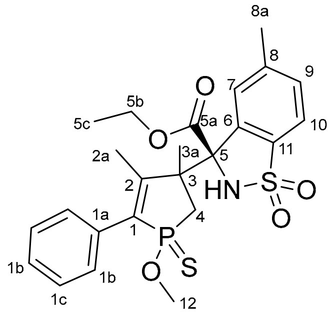

(**9b**)

NMR spectra of 8b:

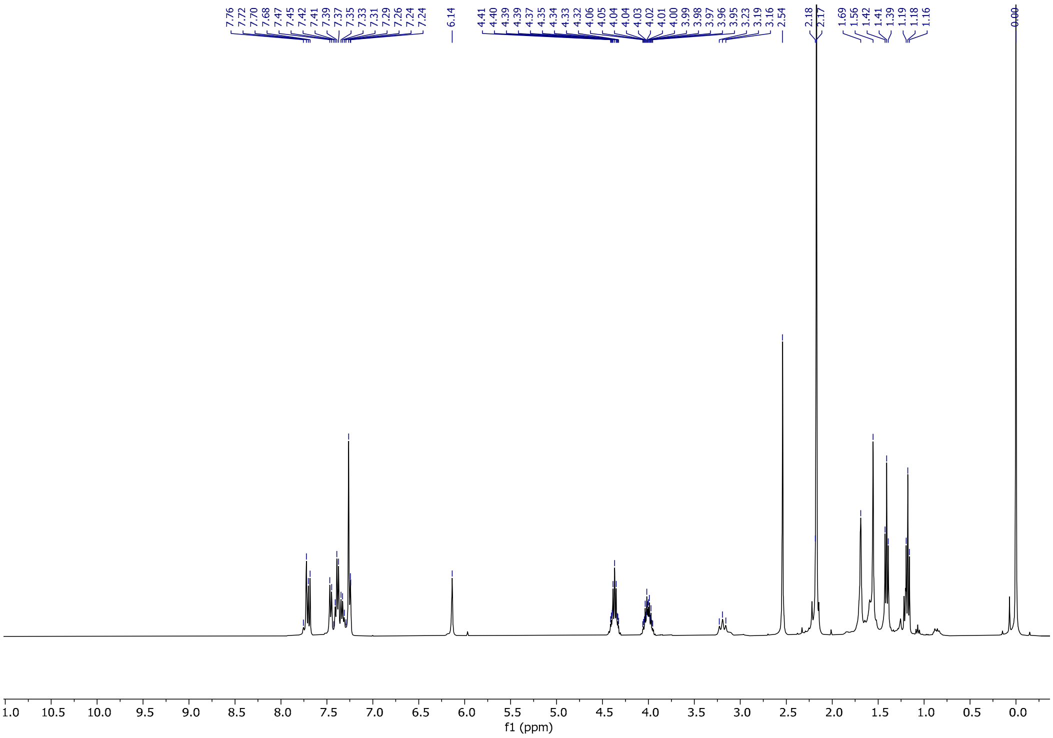

Figure S1. ^{1}H NMR spectrum of **8b** in $CDCl_3$.

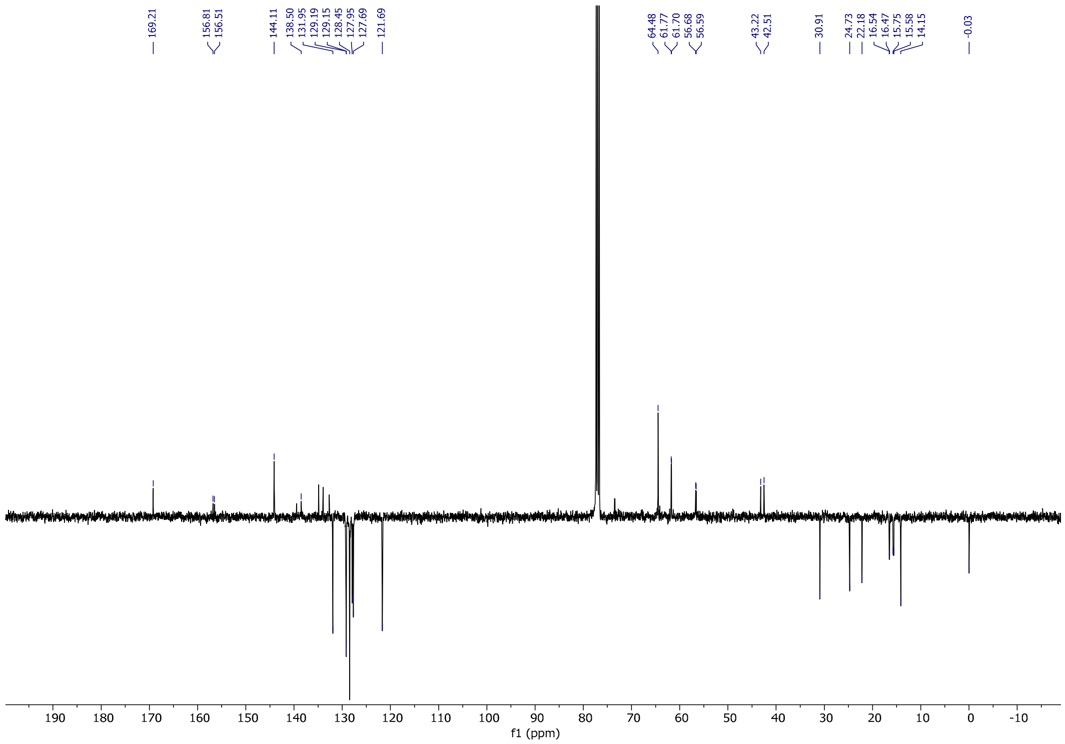

Figure S2. $^{13}C\{^1H\}$ NMR spectrum of **8b** in $CDCl_3$.

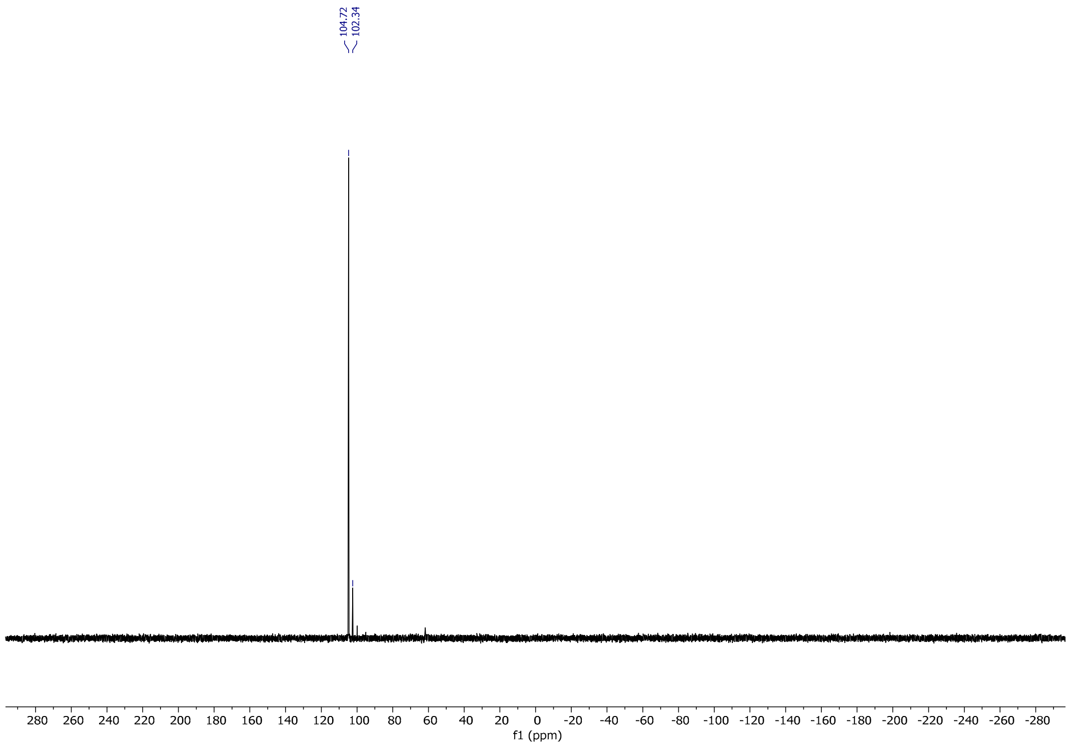

Figure S3. $^{31}P\{^1H\}$ NMR spectrum of **8b** in $CDCl_3$.

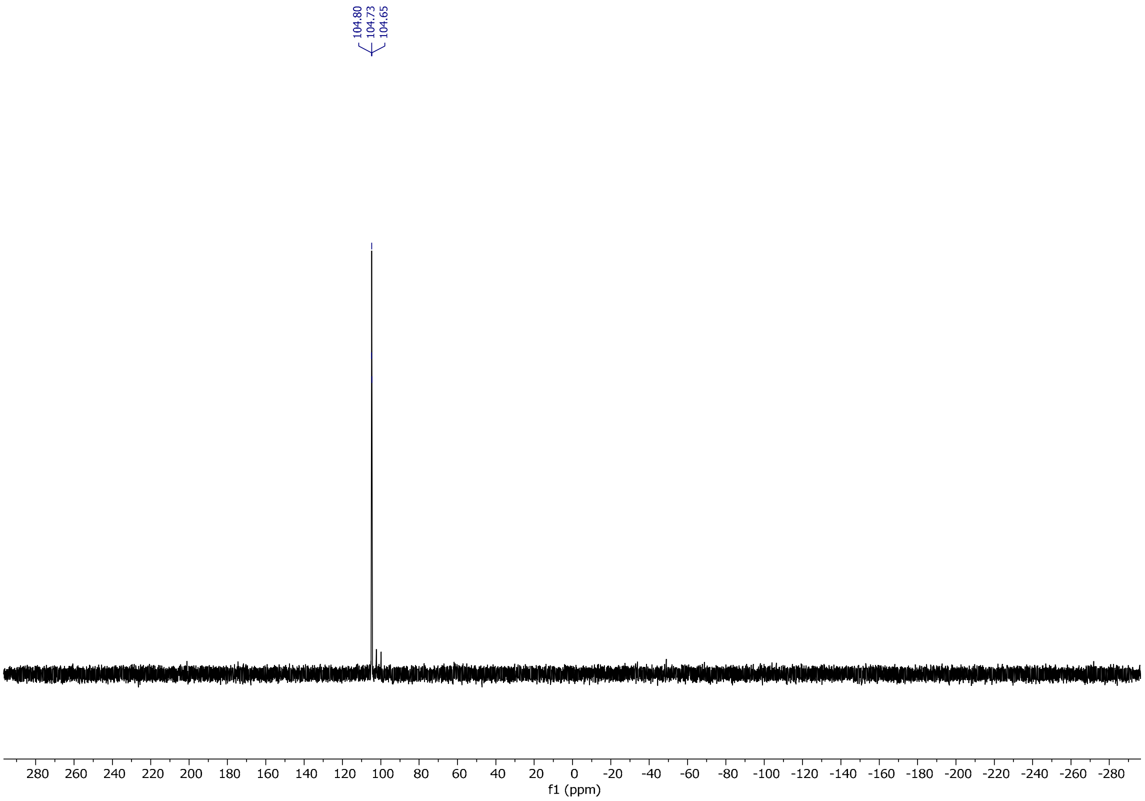

Figure S4. ^{31}P NMR spectrum of **8b** in $CDCl_3$.

9b:

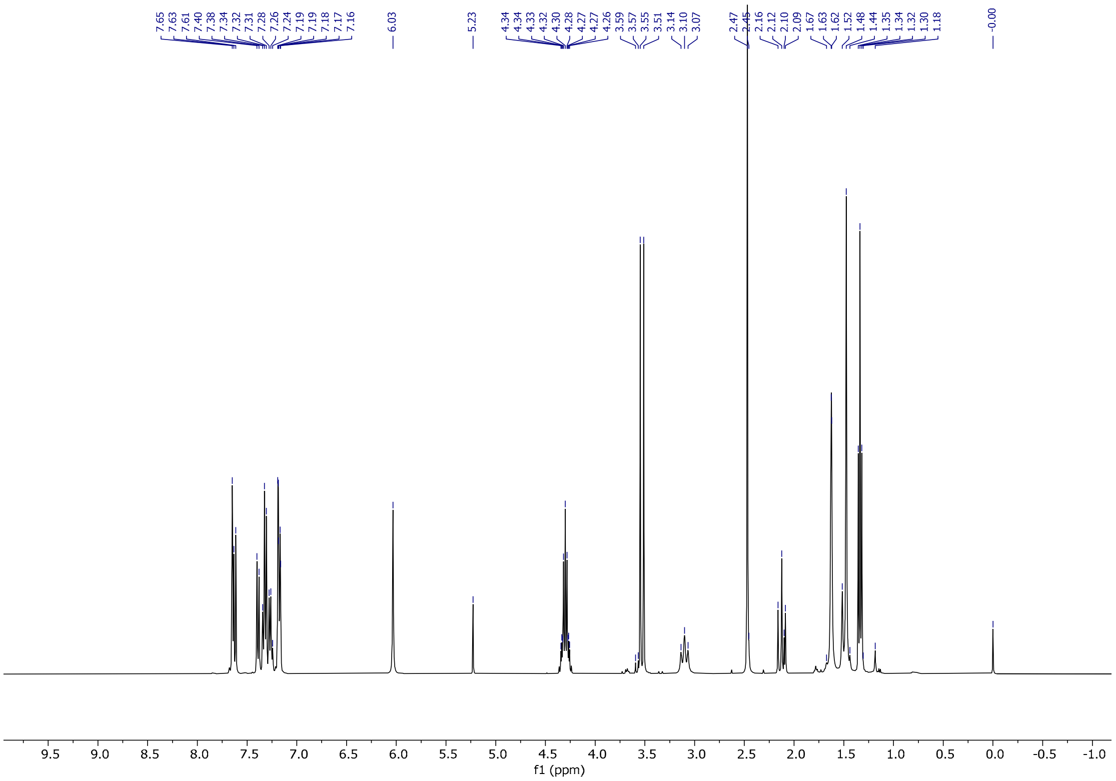

Figure S5. ^{1}H NMR spectrum of **9b** in $CDCl_3$.

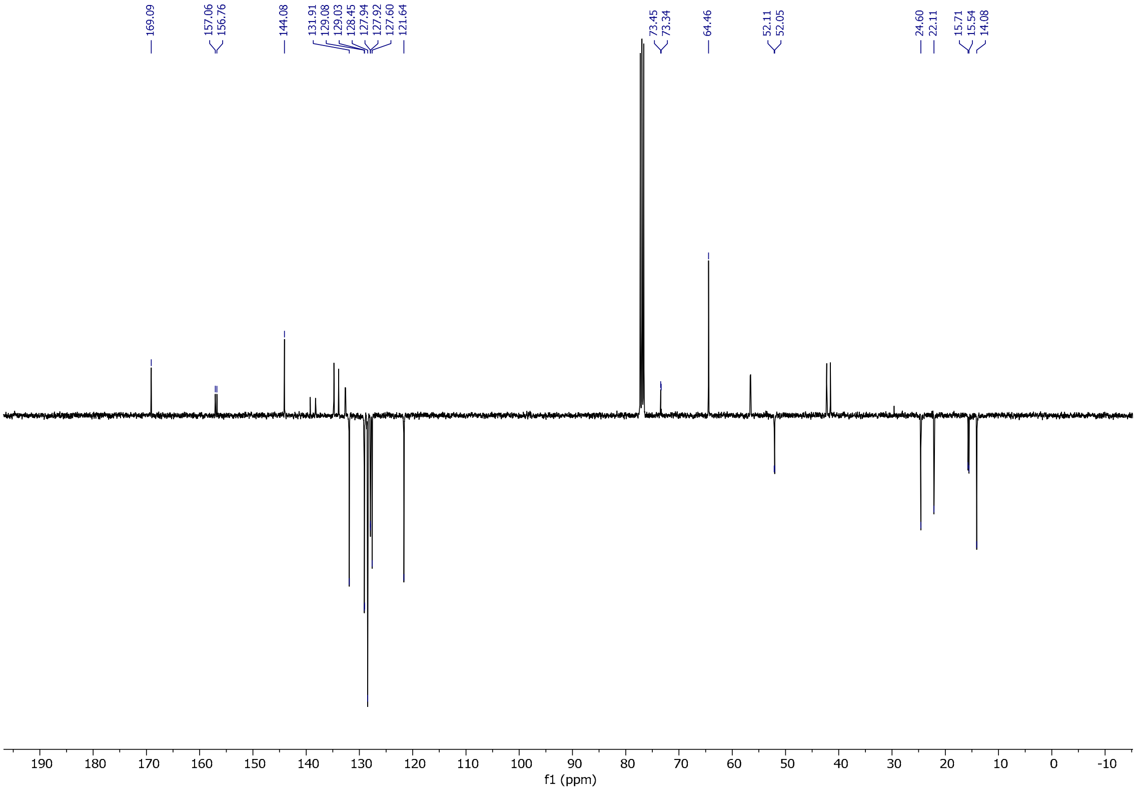

Figure S6. $^{13}C\{^{1}H\}$ NMR spectrum of **9b** in $CDCl_3$.

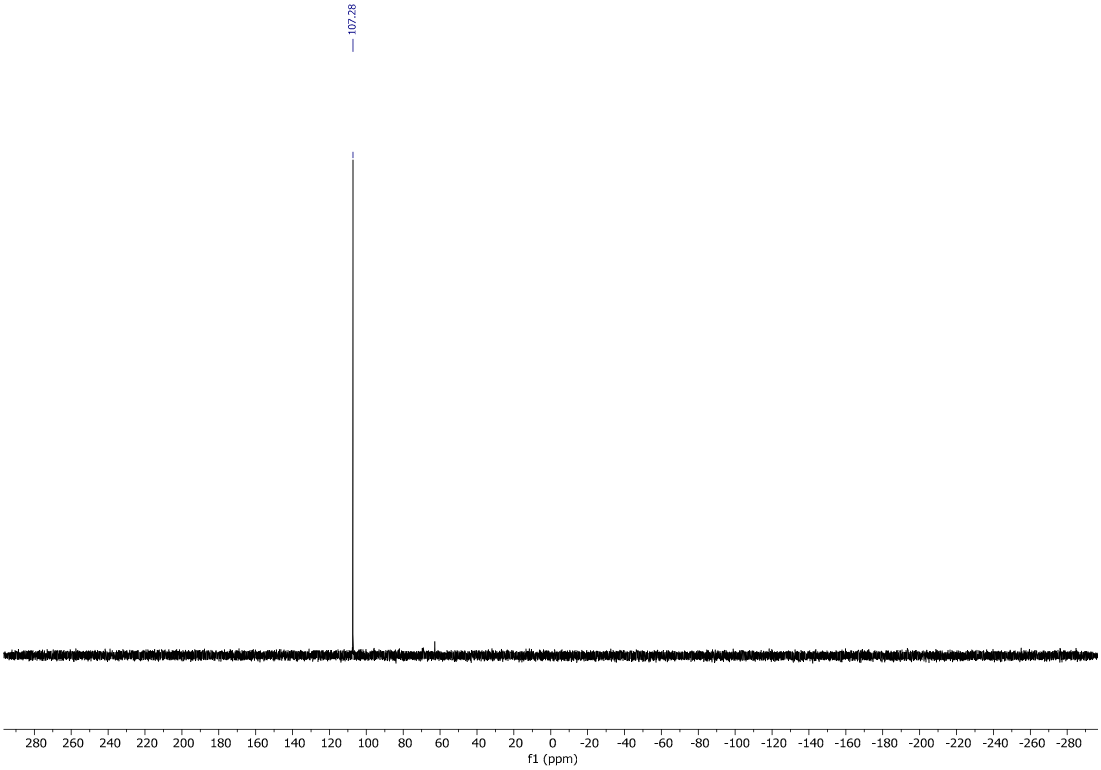

Figure S7. $^{31}P\{^{1}H\}$ NMR spectrum of **9b** in $CDCl_3$.

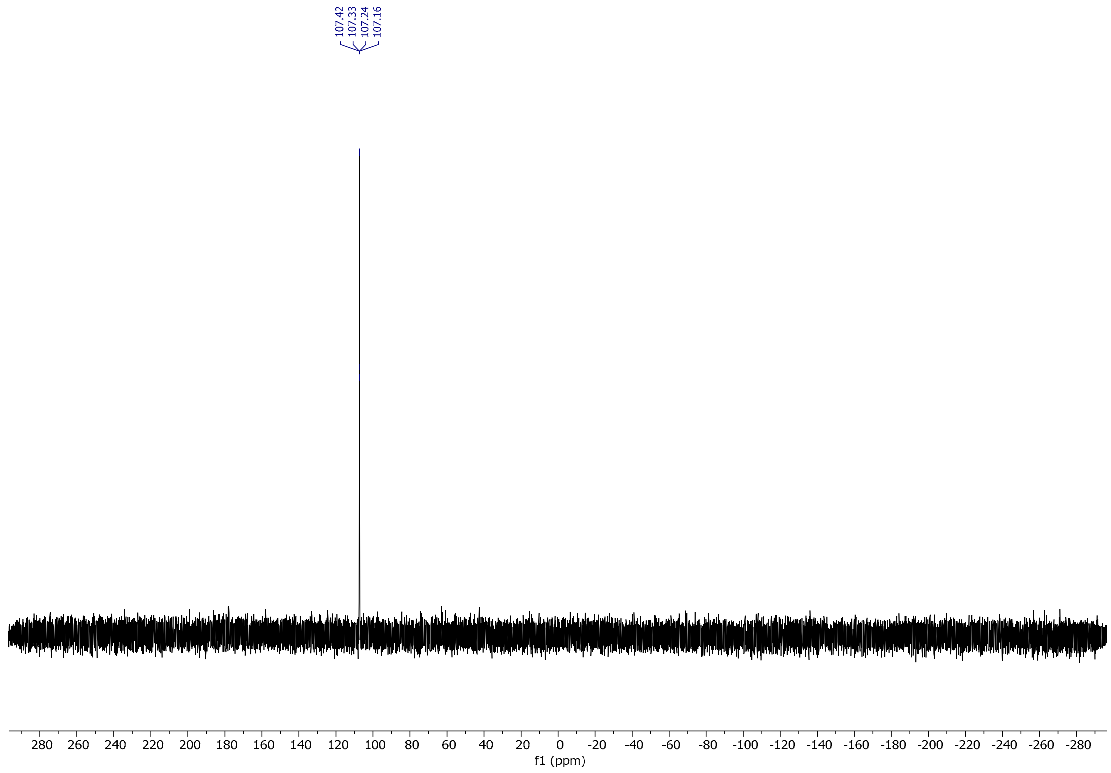

Figure S8. ^{31}P NMR spectrum of **9b** in $CDCl_3$.

2. P–N bond cleavage of *endo*-**1b** with EtOH

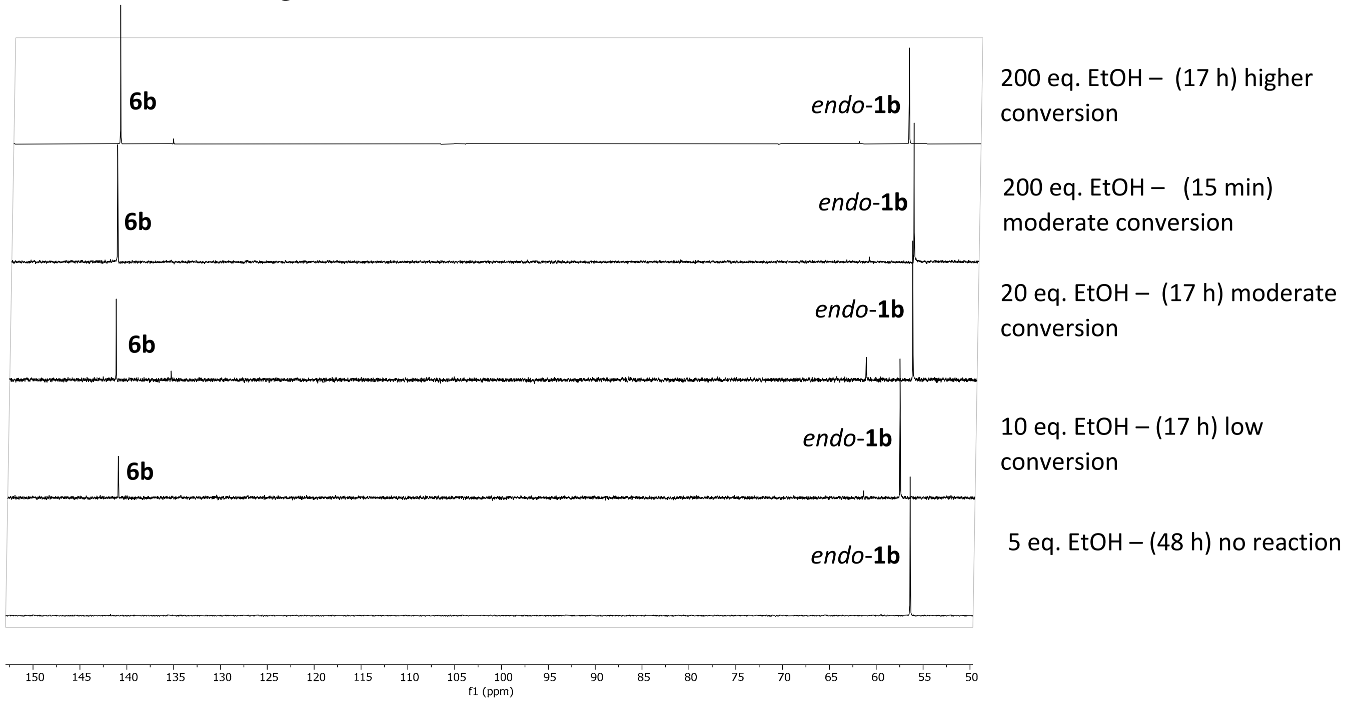

Figure S9. Time- and concentration-dependent P-N bond cleavage of *endo*-**1b** with EtOH followed by $^{31}P\{^1H\}$ NMR spectroscopy.

3.1. Reversibility studies

The reversibility experiments were conducted by adding 200 eq. of EtOH (20 eq. in the case of *endo*-**1d** was used) to a solution of the corresponding PAN in 3 ml of THF (50 mg, 0.137 mmol of *endo*-**1a** and 27 mmol, 1.6 ml of EtOH; 50 mg, 0.113 mmol of *endo*-**1b** and 23 mmol, 1.3 ml of EtOH; 50 mg, 0.113 mmol of *endo*-**1c** and 23 mmol, 1.3 ml of EtOH; 50 mg, 0.128 mmol of *endo*-**1d** and 2.6 mmol, 0.15 ml of EtOH) at room temperature. After stirring overnight, NMR samples were taken. The solutions were concentrated in vacuo followed by drying for one hour. Then, another NMR sample was taken to study the reversibility (Supplementary Materials, Figures S10-13). Only for the reaction of *endo*-**1d** with excess ethanol could product **6d** be isolated, albeit only with *endo*-**1d** as an impurity.

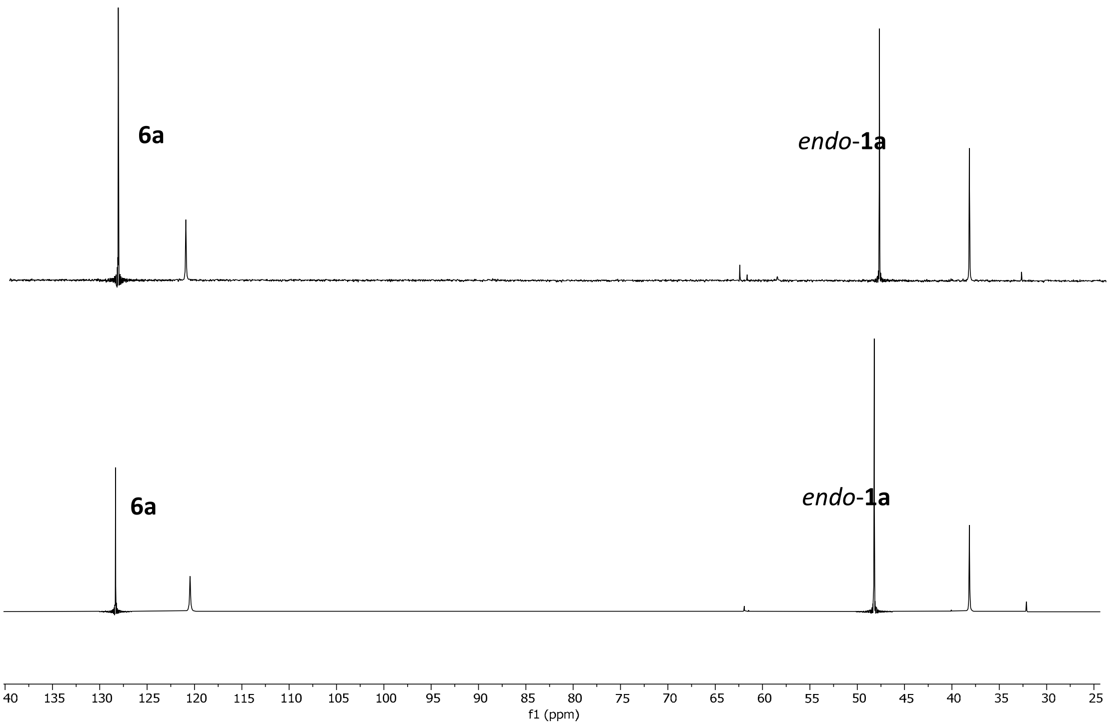

Figure S10. Ring-opening (formation of **6a**; top spectrum) and ring-closing (partial reformation of *endo*-**1a**; bottom spectrum) reaction of *endo*-**1a** followed by $^{31}P\{^{1}H\}$ NMR spectroscopy.

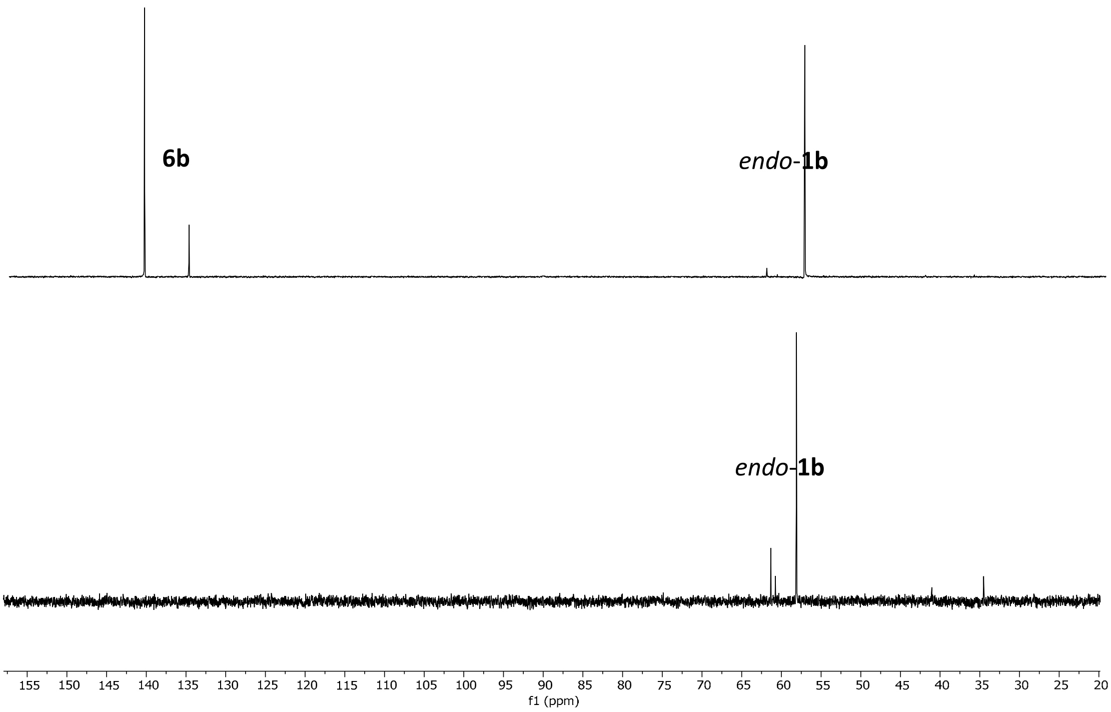

Figure S11. Ring-opening (formation of **6b**; top spectrum) and ring-closing (reformation of *endo*-**1b**; bottom spectrum) reaction of *endo*-**1b** followed by $^{31}P\{^{1}H\}$ NMR spectroscopy.

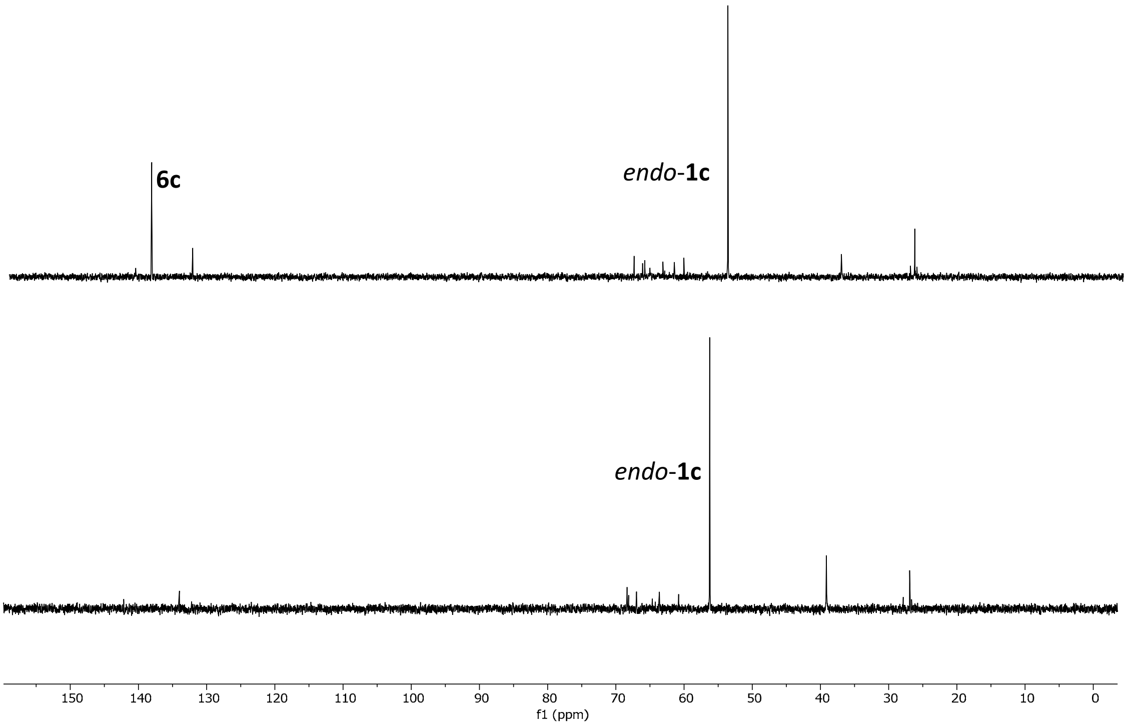

Figure S12. Ring-opening (formation of **6c**; top spectrum) and ring-closing (reformation of *endo*-**1c**; bottom spectrum) reaction of *endo*-**1c** followed by $^{31}P\{^{1}H\}$ NMR spectroscopy.

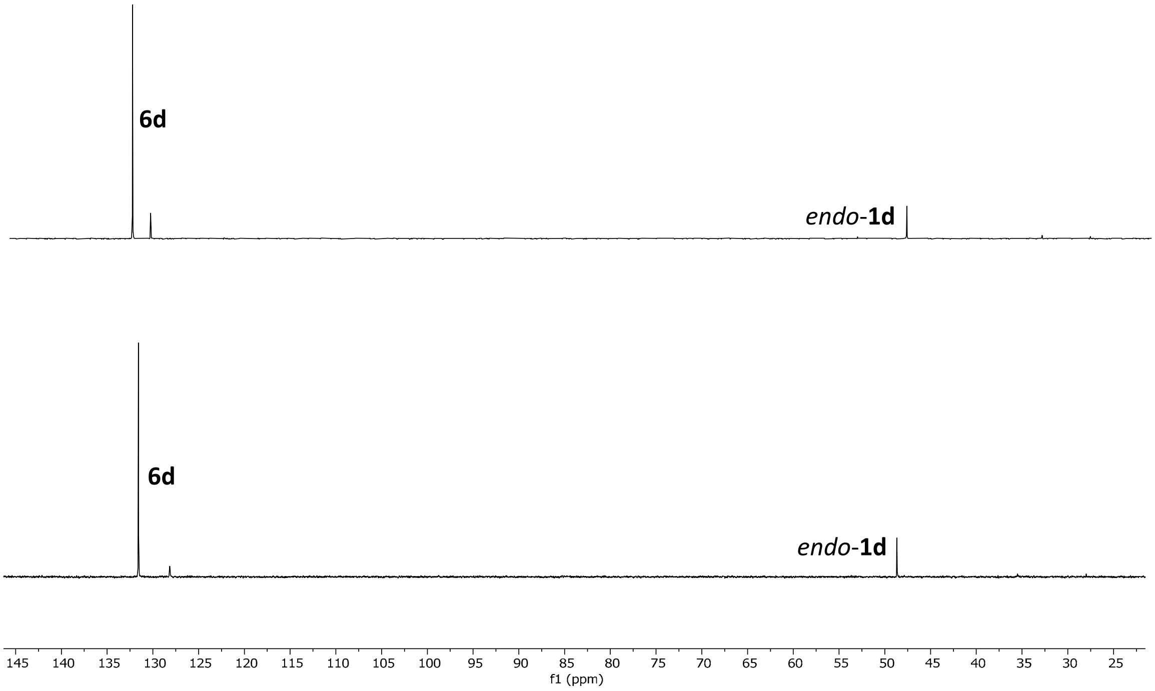

Figure S13. Ring-opening (formation of **6d**; top spectrum) and ring-closing (no reformation of *endo*-**1d**; bottom spectrum) reaction of *endo*-**1d** followed by $^{31}P\{^{1}H\}$ NMR spectroscopy.

3.2. Synthesis, NMR spectra and numbering scheme of **6d**

Reaction of *endo*-**1d** with excess ethanol:
EtOH (2.6 mmol, 0.15 mL, 20 eq.) was added to a solution of 50 mg (0.128 mmol) *endo*-**1d** dissolved in 2 mL of THF and stirred overnight at room temperature. The solvent was removed under reduced pressure to give 42 mg (75%) of **6d** as a beige oil that contains ca. 10% of *endo*-**1d** according to ^{31}P NMR spectroscopy. All attempts to isolate **6d** in pure form failed. The NMR spectra therefore also show the signals of the starting material, *endo*-**1d**.

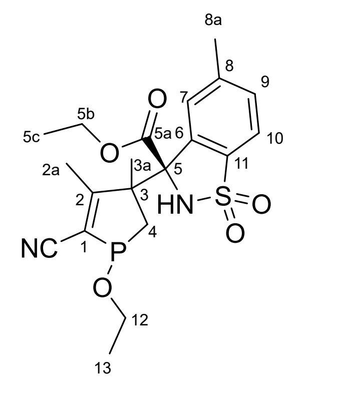

^{1}H NMR (400 MHz, THF) δ 7.61 (bs, 1H, H-7), 7.48 – 7.28 (m, 2H), 4.24 – 4.06 (m, 2H, H-5b), 3.62 (m, 2H, H-12), 2.66 – 2.39 (m, 1H, H-4), 2.37 (s, 3H, H-8a), 1.83-1.69 (m, 1H, H-4), 1.56 (s, 3H, H-2a/3a), 1.51 (s, 3H, H-2a/3a), 1.17 (t, 3H, H-5c), and 1.06 (t, *J* = 7.0 Hz, 3H, H-13) ppm; $^{13}C\{^{1}H\}$ NMR (101 MHz, THF) δ 173.3 (d, J = 2.11 Hz, CN), 167.6 (s, C-5a), 143.1 or 142.9 (s, C-quart. aryl), 135.7 (s, C-quart. aryl), 135.5 (d, *J* = 2.4 Hz) or 134.5 (s) or 134.3 (s, C-quart. aryl), 131.5 or 131.4 (s, C-aryl), 127.01 (s, C-7), 120.9 or 120.4 (s, C-aryl), 118.9 (d, *J* = 38.5 Hz, C-1/2), 115.8 (d, *J* = 23.4 Hz, C-1/2), 114.3 (d, *J* = 23.8 Hz, C-1/2), 65 (d, *J* = 19.3 Hz, C-12), 62.9 or 62.7 (s, C-quart.), 62.3 (s, C-5b), 49.1 (d, *J* = 7.0 Hz, C-4), 23.7 (s, C-methyl), 20.4 (s, C-8a), 17.1 (s, C-methyl), 16.4 (d, *J* = 24.9 Hz, C-13), and 13.1 (s, C-5c) ppm; $^{31}P\{^{1}H\}$ NMR (162 MHz, THF) δ 132.9 (s) ppm; ^{31}P NMR (162 MHz, THF) δ 132.9 (m) ppm.

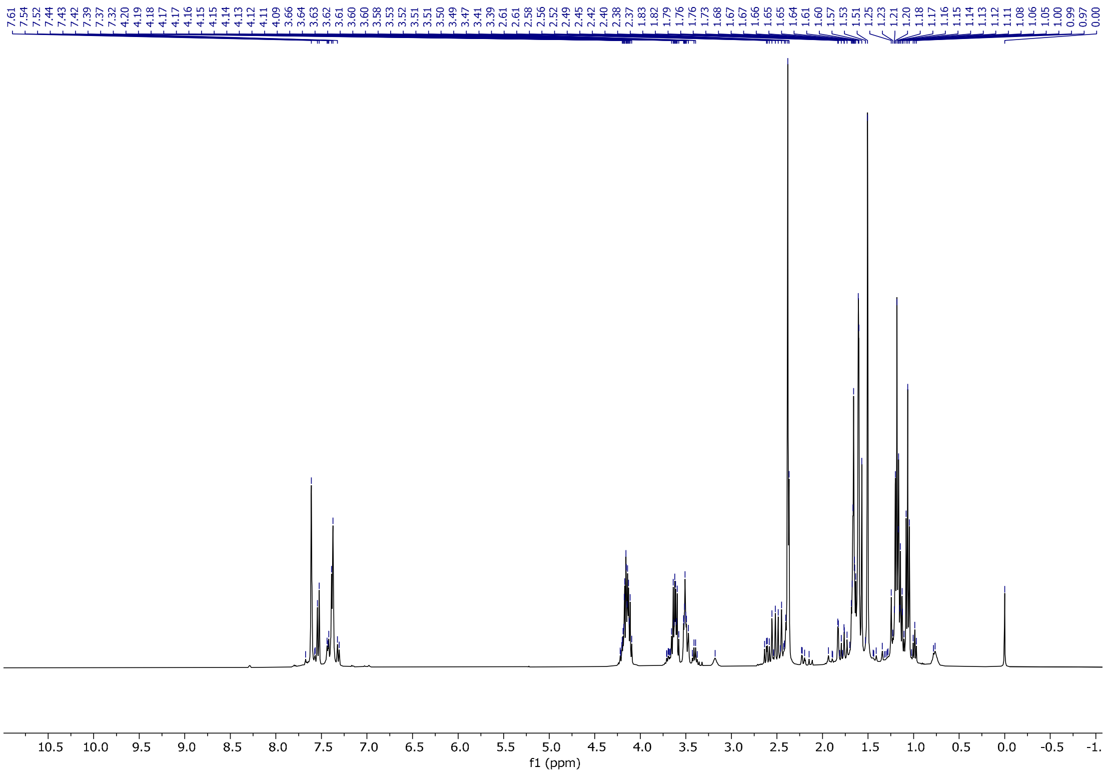

Figure S14. ^{1}H NMR spectrum of **6d** in THF-d8.

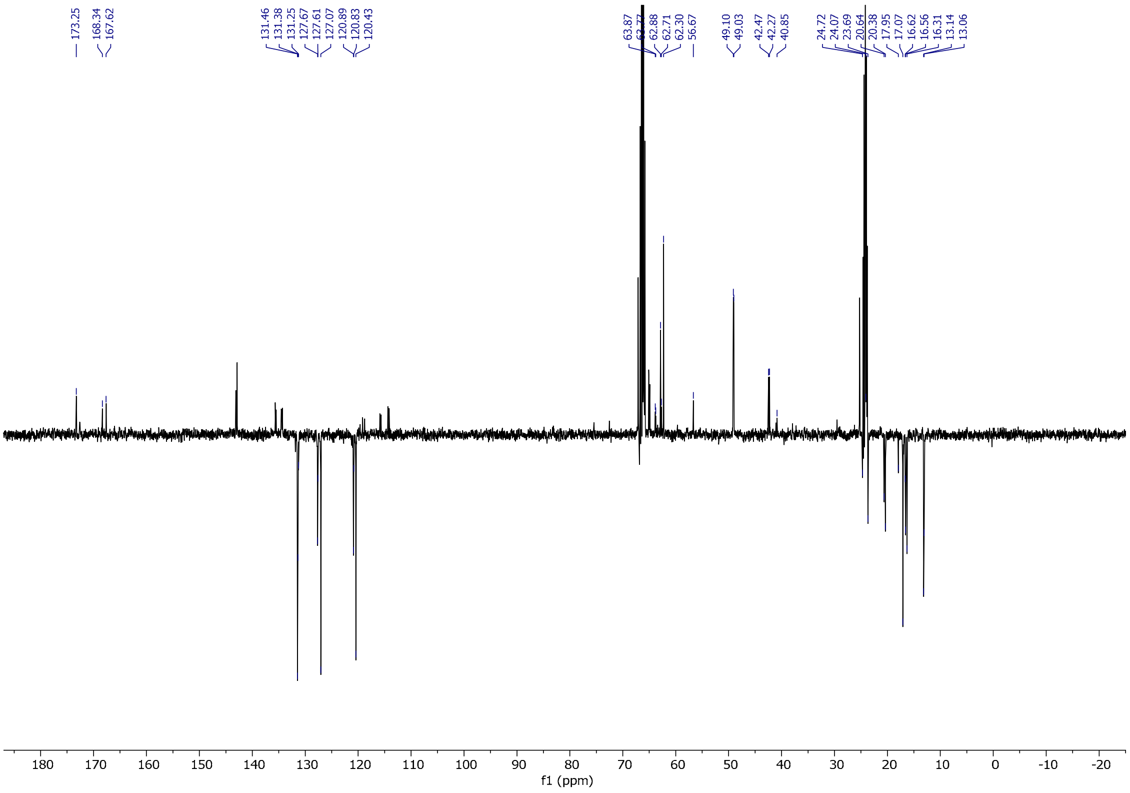

Figure S15. $^{13}C\{^{1}H\}$ NMR spectrum of **6d** in THF-d8.

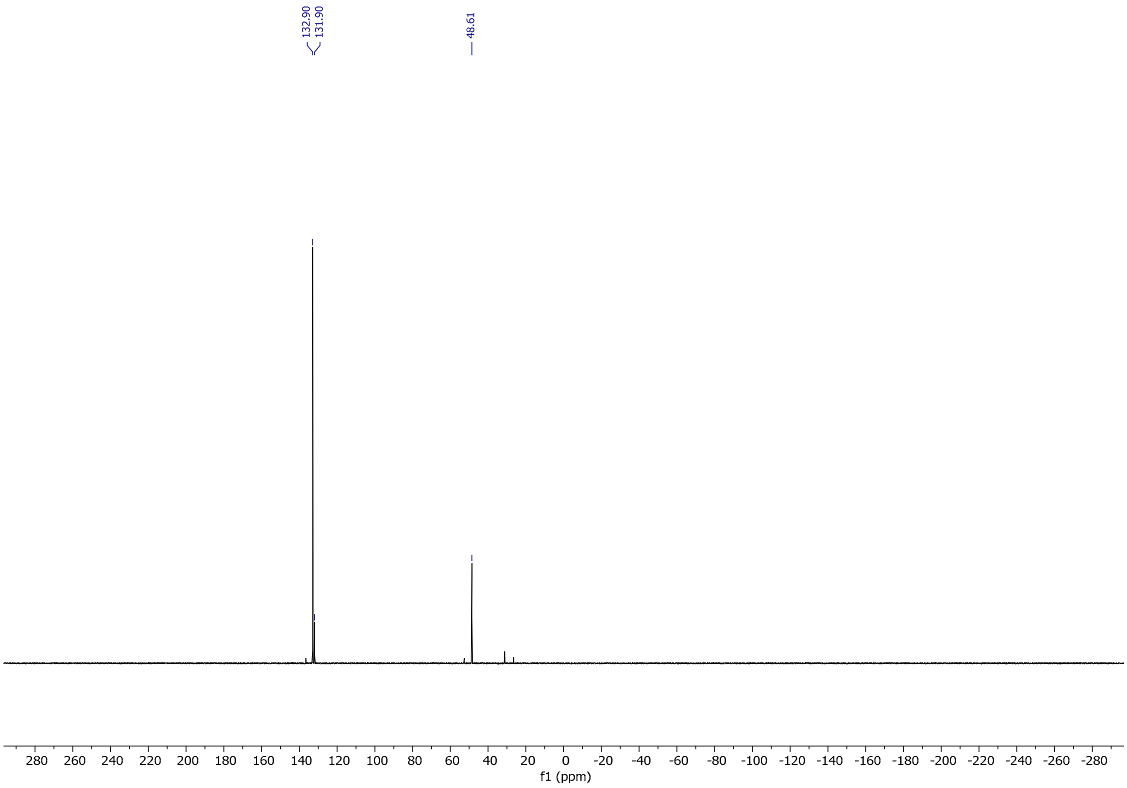

Figure S16. $^{31}P\{^{1}H\}$ NMR spectrum of **6d** in THF-d_8.

4. Theoretical calculations

4.1. xyz-structures

endo-**1a**

44

FINAL HEAT OF FORMATION = -1754.750027

S	-0.446751	-0.379560	1.942193
C	1.155520	-0.207338	1.226970
C	1.070532	-0.042094	-0.142500
C	2.239946	-0.028117	-0.892447
H	2.198993	0.051794	-1.970731
C	3.479170	-0.126982	-0.262323
C	3.527644	-0.262199	1.131590
H	4.489986	-0.342459	1.622054
C	2.368362	-0.318368	1.888538
H	2.403596	-0.453087	2.961285
C	4.751344	-0.070069	-1.062417
H	5.500454	-0.754931	-0.662779
H	4.575118	-0.326836	-2.107140
H	5.182460	0.934684	-1.035644
O	-0.708376	0.603731	2.963388
O	-0.676146	-1.763325	2.255758
N	-1.245728	0.060218	0.541522
C	-0.355178	0.049765	-0.655417
P	-2.303082	1.512579	0.598176

C	-0.938208	2.738203	0.528215
C	-0.184868	2.541802	-0.559755
C	-0.710992	1.389883	-1.427140
C	1.061974	3.292586	-0.898305
H	0.981014	3.771629	-1.877485
H	1.258011	4.064248	-0.154648
H	1.927841	2.628026	-0.938663
C	-0.245963	1.432503	-2.877117
H	-0.587673	2.360714	-3.338263
H	0.840532	1.410005	-2.961775
H	-0.659383	0.601440	-3.442724
C	-0.757926	-1.233184	-1.424083
O	-0.109927	-2.282847	-0.921351
O	-1.587634	-1.292618	-2.295164
C	-0.590108	-3.603950	-1.279191
C	-1.774431	-3.983875	-0.415240
H	-1.522216	-3.887683	0.640573
H	-2.629924	-3.343057	-0.628533
H	-2.060320	-5.017536	-0.621048
H	-0.836842	-3.611863	-2.340106
H	0.265931	-4.250964	-1.098113
C	-2.247300	1.474661	-1.249526
H	-2.636301	2.407705	-1.655612
H	-2.774390	0.627851	-1.679464
H	-0.704491	3.439225	1.317595

endo-**1b**
54
FINAL HEAT OF FORMATION = -1985.740797

S	0.472109	-0.623189	1.962142
C	0.490008	1.052829	1.412430
C	0.938588	1.119670	0.103345
C	1.185352	2.364786	-0.458634
H	1.601099	2.439648	-1.452517
C	0.914206	3.526119	0.263997
C	0.425133	3.420394	1.572083
H	0.215697	4.322467	2.133889
C	0.227492	2.184305	2.167635
H	-0.117321	2.100504	3.189378
C	1.122632	4.876954	-0.363595
H	1.823934	4.821904	-1.196250
H	0.179313	5.274190	-0.749320
H	1.506123	5.596166	0.361302
O	-0.778319	-1.027406	2.552684
O	1.685033	-0.871838	2.692233
N	0.572399	-1.234392	0.424195
C	1.142179	-0.255308	-0.524416
P	-0.760805	-2.196331	-0.255827
C	-1.821927	-0.781752	-0.839785
C	-1.094818	0.012556	-1.650379
C	0.322574	-0.518808	-1.875845
C	-1.533743	1.309296	-2.254555

H	-2.445075	1.673028	-1.784476
H	-0.763080	2.072571	-2.149478
H	-1.727396	1.198892	-3.325788
C	0.998164	-0.005009	-3.139295
H	1.181488	1.067757	-3.113276
H	1.955672	-0.497657	-3.302939
H	0.359502	-0.217294	-3.997848
C	2.648263	-0.473632	-0.765668
O	3.035351	-1.717135	-0.495822
O	3.369334	0.397570	-1.184517
C	4.455783	-1.998402	-0.588014
C	5.161080	-1.583856	0.686415
H	5.121263	-0.502669	0.815351
H	4.697184	-2.056549	1.552221
H	6.209171	-1.886696	0.639566
H	4.500845	-3.074879	-0.742376
H	4.860842	-1.485267	-1.459463
C	-3.214866	-0.593375	-0.410890
C	-3.583886	-0.776989	0.927350
H	-2.828946	-1.039282	1.654654
C	-4.900903	-0.599903	1.327894
H	-5.162918	-0.736858	2.369537
C	-5.879298	-0.252134	0.403037
H	-6.906254	-0.119116	0.718593
C	-5.530616	-0.089435	-0.932850
H	-6.287335	0.163300	-1.664950
C	-4.212768	-0.259656	-1.335634
H	-3.955394	-0.156225	-2.381446
C	0.149815	-2.054968	-1.856365
H	-0.464859	-2.390250	-2.690238
H	1.093232	-2.598469	-1.842207

endo-**1c**

53

FINAL HEAT OF FORMATION = -2001.782171

S	-0.253969	-0.505250	-1.836830
C	-0.554695	1.122379	-1.232264
C	-1.104961	1.085944	0.034713
C	-1.533945	2.272997	0.615928
H	-2.004076	2.267711	1.590063
C	-1.362289	3.483368	-0.051460
C	-0.775342	3.484442	-1.324477
H	-0.642610	4.425102	-1.844818
C	-0.378929	2.305591	-1.933176
H	0.051954	2.302217	-2.925249
C	-1.785566	4.779259	0.583694
H	-0.917103	5.404484	0.805764
H	-2.433765	5.351044	-0.083491
H	-2.323157	4.608358	1.516016
O	1.122530	-0.728996	-2.203197
O	-1.273586	-0.848287	-2.789628
N	-0.538574	-1.229794	-0.355093

C	-1.201470	-0.312036	0.618070
P	0.811920	-2.135047	0.390263
C	1.761310	-0.637657	0.943461
C	0.983756	0.098988	1.769247
C	-0.405160	-0.528736	1.974523
C	1.324054	1.395366	2.428480
H	1.068963	1.363307	3.489959
H	2.374561	1.628755	2.302190
H	0.747479	2.213480	1.987651
C	-1.142922	-0.046385	3.218093
H	-0.547172	-0.273758	4.103272
H	-1.312075	1.029790	3.209805
H	-2.099935	-0.552024	3.314290
C	-2.666156	-0.809195	0.672483
O	-3.365766	-0.269231	-0.323971
O	-3.095760	-1.624072	1.448839
C	-4.656478	-0.855881	-0.629378
C	-4.481344	-2.096470	-1.480390
H	-3.970177	-2.879656	-0.920584
H	-5.460842	-2.474061	-1.781418
H	-3.897464	-1.868086	-2.371705
H	-5.174358	-1.070664	0.304567
H	-5.181776	-0.068285	-1.166012
C	3.096607	-0.340194	0.403307
N	3.605179	0.894146	0.556068
C	4.809200	1.159218	0.060062
H	5.173133	2.170786	0.211664
C	5.583574	0.230896	-0.624337
H	6.554573	0.504845	-1.014438
C	5.063446	-1.044865	-0.794939
H	5.620524	-1.803920	-1.329330
C	3.813074	-1.334320	-0.278184
H	3.380977	-2.315138	-0.414668
C	-0.131352	-2.049691	1.967040
H	0.503370	-2.337457	2.804394
H	-1.038397	-2.647767	1.957643

endo-**1d**
45
FINAL HEAT OF FORMATION = -1846.983707

S	0.046689	-0.465883	1.933497
C	0.272942	1.135628	1.233041
C	0.536329	1.048461	-0.121842
C	0.892446	2.199632	-0.812828
H	1.153331	2.152273	-1.861759
C	0.920270	3.430400	-0.158664
C	0.609422	3.485432	1.206715
H	0.628073	4.441394	1.715217
C	0.299549	2.339008	1.920112
H	0.085782	2.376341	2.979761
C	1.255409	4.689454	-0.909290
H	0.351642	5.269341	-1.115793

H	1.923609	5.328321	-0.329843
H	1.734338	4.469122	-1.863179
O	-1.203118	-0.609713	2.631373
O	1.258254	-0.861060	2.595200
N	-0.087958	-1.214363	0.439215
C	0.420370	-0.361454	-0.674913
P	-1.614806	-2.037746	0.044087
C	-2.553089	-0.490457	-0.392884
C	-1.914209	0.185948	-1.369552
C	-0.650732	-0.542834	-1.831556
C	-2.337647	1.499427	-1.928949
H	-3.259688	1.843204	-1.463586
H	-1.565919	2.255579	-1.771222
H	-2.496050	1.426244	-3.007776
C	-0.169716	-0.131740	-3.217761
H	-0.957347	-0.326250	-3.947284
H	0.069186	0.930053	-3.273423
H	0.707736	-0.704108	-3.505330
C	1.803360	-0.964116	-1.023130
O	2.730081	-0.460005	-0.215040
O	1.995804	-1.821824	-1.846784
C	4.011195	-1.142002	-0.151914
C	3.913078	-2.366382	0.733332
H	3.515096	-2.097725	1.711444
H	3.263516	-3.118507	0.286011
H	4.905236	-2.804259	0.860262
H	4.324580	-1.389993	-1.165285
H	4.681618	-0.393755	0.265510
C	-1.029223	-2.040142	-1.707736
H	-1.845824	-2.297527	-2.381356
H	-0.184550	-2.703891	-1.869726
C	-3.716378	-0.040430	0.272050
N	-4.679552	0.308842	0.803520

6a

53

FINAL HEAT OF FORMATION = -1909.780419

S	-1.691248	-1.667670	0.166278
C	-2.388484	-0.121961	-0.314814
C	-1.495396	0.918119	-0.123082
C	-1.894594	2.209384	-0.457577
H	-1.233853	3.044492	-0.285374
C	-3.157852	2.438635	-0.999048
C	-4.024632	1.356301	-1.199991
H	-5.006478	1.533097	-1.621924
C	-3.649825	0.068321	-0.857985
H	-4.318414	-0.769330	-1.004628
C	-3.595381	3.832209	-1.358771
H	-4.460066	4.137261	-0.764519
H	-2.797578	4.554796	-1.189018
H	-3.888185	3.891917	-2.409380
O	-1.433494	-2.449267	-1.009611

O	-2.391932	-2.282827	1.263165
N	-0.294673	-0.968406	0.762316
C	-0.153378	0.462694	0.456688
P	2.394473	-1.810470	-0.247621
C	1.482170	-1.401493	-1.761343
C	0.887671	-0.208066	-1.775300
C	1.107263	0.636927	-0.517302
C	0.060561	0.295792	-2.920813
H	-0.022475	-0.469912	-3.690497
H	-0.948199	0.574059	-2.613843
H	0.512116	1.184247	-3.370425
C	1.412124	2.091573	-0.895854
H	0.564042	2.590799	-1.360422
H	1.726255	2.676468	-0.035290
H	2.228570	2.088996	-1.619326
C	0.072799	1.158111	1.815511
O	0.126611	2.483695	1.709578
O	0.175658	0.555715	2.855321
C	0.253960	3.245064	2.940322
C	-1.100814	3.462718	3.579706
H	-0.990704	4.096179	4.462005
H	-1.782127	3.956399	2.885394
H	-1.538680	2.515199	3.892227
H	0.933758	2.717246	3.606744
H	0.707733	4.182209	2.623285
C	2.320831	-0.003560	0.201167
H	3.241201	0.429799	-0.193039
H	2.311050	0.155855	1.279038
H	1.368200	-2.114255	-2.568643
O	3.959832	-1.869337	-0.828484
C	4.750744	-3.007263	-0.475950
C	4.564683	-4.148197	-1.458950
H	5.224209	-4.981911	-1.205549
H	3.534403	-4.509628	-1.437279
H	4.794154	-3.819706	-2.474064
H	4.500304	-3.335417	0.539049
H	5.787633	-2.664341	-0.472996
H	-0.219091	-1.140550	1.761203

6b

63

FINAL HEAT OF FORMATION = -2140.749589

S	-0.365327	-1.359622	1.716505
C	-1.795093	-1.839107	0.781888
C	-2.207056	-0.774020	0.002864
C	-3.344487	-0.914991	-0.780429
H	-3.706730	-0.086245	-1.366108
C	-4.031792	-2.125244	-0.795940
C	-3.575385	-3.186880	-0.014527
H	-4.110766	-4.123379	-0.028977
C	-2.454881	-3.049354	0.787125
H	-2.096480	-3.853905	1.408140

C	-5.256555	-2.294111	-1.639115
H	-6.109844	-2.547547	-1.012938
H	-5.481549	-1.385226	-2.188757
H	-5.113361	-3.108029	-2.347325
O	0.811639	-2.024943	1.236288
O	-0.586084	-1.419351	3.138165
N	-0.404695	0.256612	1.231115
C	-1.345696	0.466346	0.142378
P	1.709792	1.562396	0.273340
C	1.729987	-0.018477	-0.673551
C	0.568775	-0.277248	-1.287635
C	-0.486027	0.803117	-1.134315
C	0.251286	-1.532908	-2.034948
H	0.394730	-1.392725	-3.106622
H	0.902723	-2.334285	-1.694735
H	-0.781180	-1.830551	-1.868019
C	-1.296467	0.971877	-2.416138
H	-0.609416	1.200911	-3.227860
H	-1.832968	0.063836	-2.669730
H	-2.003706	1.789652	-2.323864
C	-2.264783	1.626852	0.561311
O	-3.128767	1.988081	-0.362990
O	-2.209234	2.112283	1.668469
C	-4.045644	3.055282	-0.045952
C	-3.412871	4.418512	-0.278696
H	-3.061789	4.504615	-1.303927
H	-4.150692	5.192155	-0.089378
H	-2.573168	4.563790	0.397186
H	-4.891724	2.893364	-0.714897
H	-4.359062	2.949246	0.996250
C	2.937952	-0.828241	-0.619847
C	3.649663	-0.893306	0.577291
H	3.256432	-0.375797	1.442863
C	4.813924	-1.630018	0.670122
H	5.345661	-1.679794	1.609663
C	5.303851	-2.298096	-0.441754
H	6.217179	-2.870741	-0.371599
C	4.621459	-2.218070	-1.644061
H	5.006648	-2.724543	-2.517701
C	3.448533	-1.490107	-1.735523
H	2.938597	-1.411504	-2.683221
C	0.288377	2.085476	-0.802506
H	0.725425	2.515140	-1.706919
H	-0.337957	2.838688	-0.323667
O	2.836188	2.416797	-0.619841
C	4.162637	2.621570	-0.161816
C	4.230625	3.279915	1.208103
H	5.266499	3.467877	1.475728
H	3.683193	4.219280	1.197828
H	3.781748	2.624086	1.952030
H	4.703994	1.667694	-0.147504
H	4.619744	3.268676	-0.918385

H	-0.631078	0.846819	2.029020

6c

62

FINAL HEAT OF FORMATION = -2156.809588

S	1.386966	1.678802	1.310127
C	2.737583	0.974318	0.423997
C	2.468019	-0.332742	0.057775
C	3.433235	-1.038186	-0.655863
H	3.265631	-2.070815	-0.921238
C	4.631832	-0.423902	-1.014261
C	4.856995	0.909268	-0.646402
H	5.788947	1.386729	-0.923756
C	3.914310	1.619623	0.077426
H	4.087036	2.646036	0.372128
C	5.679019	-1.180238	-1.785044
H	5.877631	-0.701352	-2.746727
H	6.623861	-1.210019	-1.237573
H	5.366441	-2.206130	-1.977384
O	0.717946	2.616031	0.452957
O	1.741016	2.091222	2.641479
N	0.556627	0.230397	1.406803
C	1.097108	-0.823345	0.535638
P	-2.309720	-0.251102	0.572520
C	-1.569700	0.701716	-0.817788
C	-0.402393	0.205939	-1.265021
C	0.025793	-1.117135	-0.623426
C	0.498943	0.850276	-2.278577
H	0.452206	1.934932	-2.203820
H	1.536069	0.551903	-2.141424
H	0.220991	0.561885	-3.297382
C	0.531418	-2.079264	-1.707189
H	0.732132	-3.068730	-1.305502
H	-0.245061	-2.172062	-2.467561
H	1.431846	-1.717569	-2.199886
C	1.292710	-2.054514	1.444621
O	1.779013	-3.105461	0.785837
O	1.040165	-2.051927	2.623513
C	1.965664	-4.332937	1.540717
C	0.673231	-5.116500	1.631175
H	-0.074162	-4.569704	2.205047
H	0.274417	-5.325238	0.637610
H	0.858508	-6.068178	2.132991
H	2.725828	-4.871967	0.978206
H	2.353116	-4.078892	2.525764
C	-2.262997	1.929359	-1.219907
N	-3.103289	2.430065	-0.296258
C	-3.774789	3.542254	-0.566098
H	-4.430028	3.906790	0.218819
C	-3.680176	4.220702	-1.775554
H	-4.250054	5.124162	-1.946285
C	-2.852475	3.686842	-2.753448

H	-2.765184	4.162044	-3.722602
C	-2.138769	2.531022	-2.478980
H	-1.513766	2.089746	-3.238567
C	-1.245771	-1.680105	0.049414
H	-1.827978	-2.228481	-0.692721
H	-1.032538	-2.363458	0.870725
O	-3.734452	-0.762809	-0.123626
C	-4.956007	-0.409116	0.534092
C	-5.408292	-1.510697	1.473858
H	-4.678847	-1.652805	2.274201
H	-6.369509	-1.257458	1.928359
H	-5.517339	-2.453711	0.934737
H	-4.831019	0.536250	1.068285
H	-5.690091	-0.247620	-0.258596
H	0.507908	-0.079036	2.373883

6d

54

FINAL HEAT OF FORMATION = -2002.016125

S	-0.451603	1.817266	-1.683483
C	-1.867680	1.761069	-0.632785
C	-1.921184	0.569984	0.074231
C	-2.978476	0.360316	0.951671
H	-3.067860	-0.575678	1.481769
C	-3.950316	1.345698	1.132269
C	-3.852090	2.542735	0.414879
H	-4.609660	3.304846	0.548013
C	-2.810617	2.762458	-0.474309
H	-2.739044	3.682887	-1.037874
C	-5.080873	1.122577	2.099263
H	-5.949940	1.727891	1.842131
H	-5.385198	0.075303	2.117930
H	-4.780064	1.393742	3.115106
O	0.505500	2.724711	-1.119376
O	-0.786669	1.931213	-3.076913
N	-0.029128	0.223228	-1.375784
C	-0.770196	-0.383168	-0.260929
P	2.697412	-0.621625	-0.419919
C	2.205817	0.763827	0.694264
C	0.996403	0.643583	1.265911
C	0.264267	-0.655494	0.929967
C	0.393859	1.671852	2.168134
H	-0.619998	1.933009	1.863868
H	0.333386	1.294135	3.192411
H	0.994106	2.579150	2.172689
C	-0.374162	-1.232858	2.200303
H	-1.163431	-0.595978	2.594270
H	-0.784466	-2.223654	2.027583
H	0.398420	-1.313611	2.965941
C	-1.362673	-1.696634	-0.821030
O	-2.075090	-2.365417	0.081359
O	-1.200769	-2.055153	-1.960678

C	-2.726539	-3.590009	-0.358389
C	-4.055253	-3.290878	-1.017854
H	-4.705851	-2.731262	-0.344380
H	-3.915827	-2.717871	-1.933782
H	-4.551931	-4.228727	-1.273765
H	-2.052083	-4.117790	-1.030186
H	-2.849661	-4.162680	0.558827
C	1.349265	-1.615096	0.382686
H	1.828841	-2.130241	1.216104
H	0.947494	-2.371658	-0.290244
C	3.061451	1.878441	0.869373
N	3.809405	2.747376	1.002494
O	3.967210	-1.286780	0.414411
C	5.294807	-1.052759	-0.080815
C	6.181052	-2.200753	0.349876
H	7.205269	-2.032636	0.010105
H	6.189714	-2.291892	1.437075
H	5.824914	-3.141685	-0.072957
H	5.268475	-0.963633	-1.172048
H	5.659288	-0.104161	0.323379
H	-0.135737	-0.327358	-2.224230

4.2. HOMO and LUMO of **6a-d**

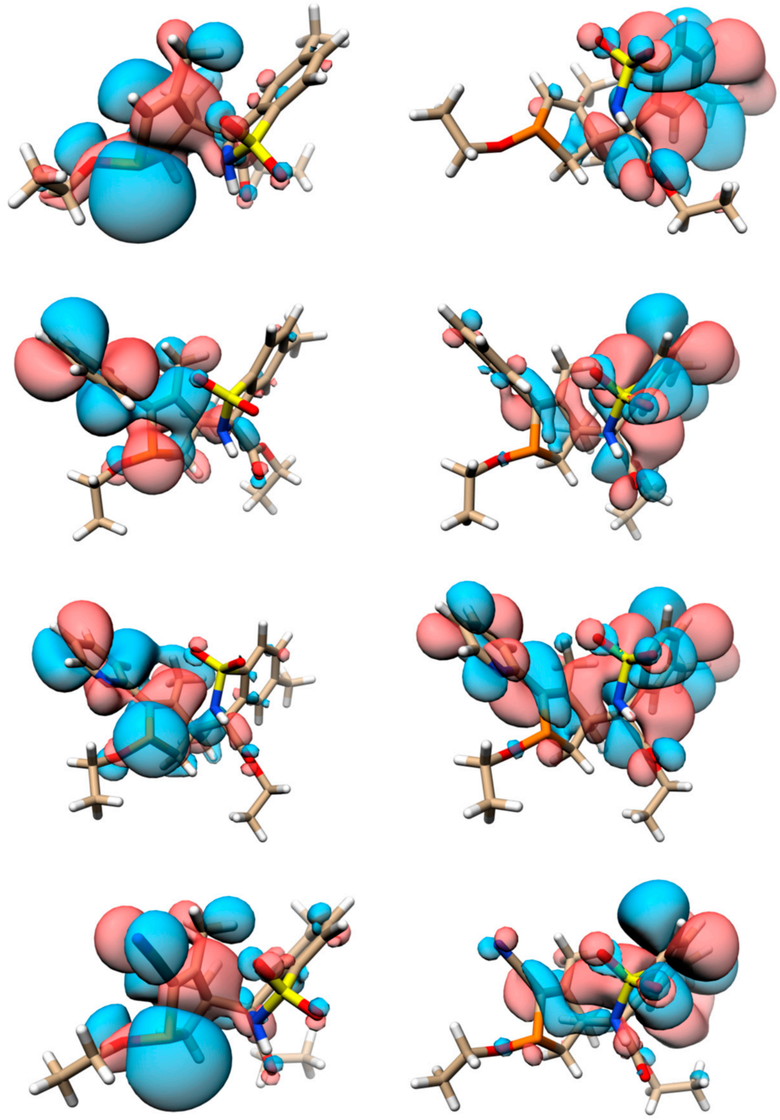

HOMO (left) and LUMO (right) of **6a**

HOMO (left) and LUMO (right) of **6b**

HOMO (left) and LUMO (right) of **6c**

HOMO (left) and LUMO (right) of **6d**

Figure S17. HOMO and LUMO of **6a-d**.

5. X-ray crystallography data

Table S1. Fundamental structure parameters.

Compound	**8b**	**9b**
Empirical formula	$C_{25}H_{30}NO_5PS_2$	$C_{24}H_{28}NO_5PS_2$
Formula weight	519.59	505.56
Temperature (K)	130(2)	130(2)
Wavelength (pm)	71.073	71.073
Crystal system	Orthorhombic	Monoclinic
Space group	Pca2$_1$	I2/a
Unit cell dimensions		
a (pm)	1582.76(5)	1367.65(5)
b (pm)	779.70(2)	803.07(4)
c (pm)	4061.3(1)	4374.7(2)
α (deg)	90	90
β (deg)	90	94.635(4)
γ (deg)	90	90
Volume (nm^3)	5.0119(3)	4.7891(4)
Z	8	8
$\rho_{(calculated)}$ (Mg m^{-3})	1.377	1.402
μ (mm^{-1})	0.313	0.326
F(000)	2192	2128
Crystal size (mm^3)	0.30 · 0.10 · 0.01	0.20 · 0.15 · 0.05
Θ_{Min} / Θ_{Max} (deg)	2.006 / 26.957	1.868 / 28.328
Index ranges	$-20 \leq h \leq 16$<br>$-9 \leq k \leq 9$<br>$-51 \leq l \leq 40$	$-18 \leq h \leq 18$<br>$-7 \leq k \leq 9$<br>$-56 \leq l \leq 55$
Reflections collected	33257	18896
Indp. reflections (R_{int})	9212 (0.0922)	5156 (0.0625)
Completeness (Θ)	100.0 % (25.35°)	100.0 % (25.35°)
T_{Max} / T_{Min}	1.00000 / 0.57738	1.00000 / 0.83668
Restraints / parameters	2 / 630	15 / 335
Gof on F^2	1.065	1.030
R1 / wR2 ($I>2\sigma(I)$)	0.0628, 0.1203	0.0572, 0.1275
R1 / wR2 (all data)	0.1012, 0.1392	0.1009, 0.1490
Residual electron density (e·$Å^{-3}$)	0.380 / -0.333	0.540 / -0.403
Comments	0.55(14) : 0.45(14) †1	-
CCDC No	2291772	2291773

†1: Refined as racemic twin.

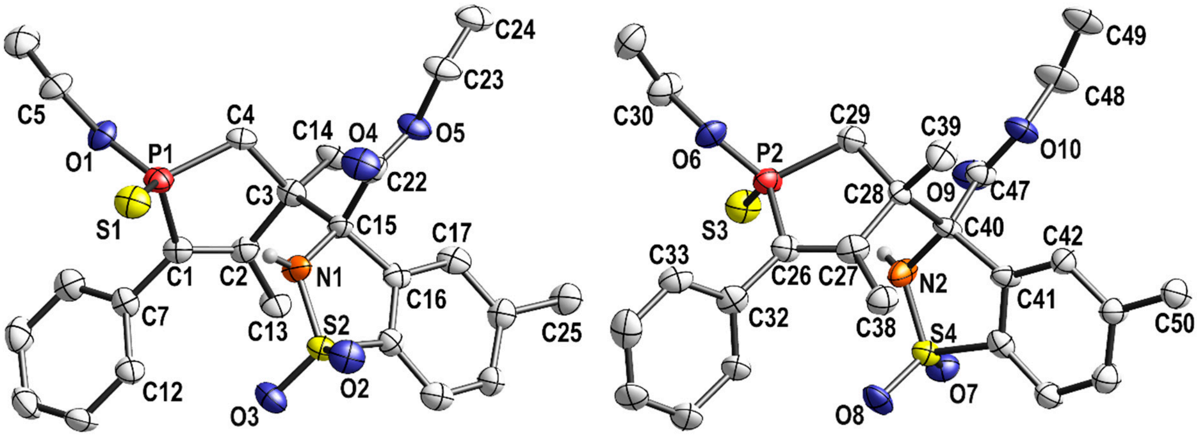

Figure S18. Molecular structure of **8b**. Except NH, all hydrogen atoms were omitted for clarity. Both independent molecules of **8b** are shown. Displacement ellipsoids are drawn at the 50 % probability level.

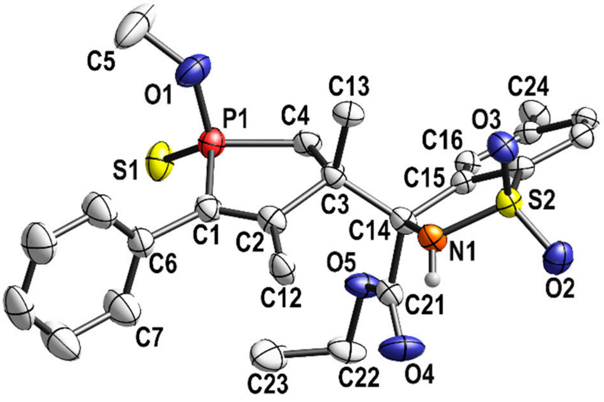

Figure S19. Molecular structure of **9b**. Except NH, all hydrogen atoms and the minor 13% disordered S=P-O-Me fraction were omitted for clarity. Displacement ellipsoids are drawn at the 50 % probability level.
